# ETV7 reduces inflammatory responses in breast cancer cells by repressing the TNFR1/NF-κB axis

**DOI:** 10.1038/s41419-023-05718-y

**Published:** 2023-04-12

**Authors:** Erna Marija Meškytė, Laura Pezzè, Laura Bartolomei, Mattia Forcato, Irene Adelaide Bocci, Giovanni Bertalot, Mattia Barbareschi, Leticia Oliveira-Ferrer, Alessandra Bisio, Silvio Bicciato, Daiva Baltriukienė, Yari Ciribilli

**Affiliations:** 1 Laboratory of Molecular Cancer Genetics, Department of Cellular, Computational, and Integrative Biology (CIBIO), University of Trento, Trento, Italy; 2 Department of Biological Models, Institute of Biochemistry, Life Sciences Center, Vilnius University, Vilnius, Lithuania; 3 Alia Therapeutics, s.r.l., Trento, Italy; 4 Laboratory of Radiobiology, Department of Cellular, Computational, and Integrative Biology (CIBIO), University of Trento, Trento, Italy; 5 Department of Life Sciences, University of Modena and Reggio Emilia, Modena, Italy; 6 Unità Operativa Multizonale di Anatomia Patologica, APSS, Trento, Italy; 7 Centre for Medical Sciences (CISMed), University of Trento, Trento, Italy; 8 Gynecology Department, University Medical Center Hamburg-Eppendorf, Hamburg, Germany; 9 Present Address: Institut für Zellbiologie, Universitätsklinikum Essen, Essen, Germany

**Keywords:** Breast cancer, Transcription

## Abstract

The transcription factor ETV7 is an oncoprotein that is up-regulated in all breast cancer (BC) types. We have recently demonstrated that ETV7 promoted breast cancer progression by increasing cancer cell proliferation and stemness and was also involved in the development of chemo- and radio-resistance. However, the roles of ETV7 in breast cancer inflammation have yet to be studied. Gene ontology analysis previously performed on BC cells stably over-expressing ETV7 demonstrated that ETV7 was involved in the suppression of innate immune and inflammatory responses. To better decipher the involvement of ETV7 in these signaling pathways, in this study, we identified *TNFRSF1A*, encoding for the main receptor of TNF-α, TNFR1, as one of the genes down-regulated by ETV7. We demonstrated that ETV7 directly binds to the intron I of this gene, and we showed that the ETV7-mediated down-regulation of TNFRSF1A reduced the activation of NF-κB signaling. Furthermore, in this study, we unveiled a potential crosstalk between ETV7 and STAT3, another master regulator of inflammation. While it is known that STAT3 directly up-regulates the expression of TNFRSF1A, here we demonstrated that ETV7 reduces the ability of STAT3 to bind to the *TNFRSF1A* gene via a competitive mechanism, recruiting repressive chromatin remodelers, which results in the repression of its transcription. The inverse correlation between ETV7 and TNFRSF1A was confirmed also in different cohorts of BC patients. These results suggest that ETV7 can reduce the inflammatory responses in breast cancer through the down-regulation of TNFRSF1A.

## Introduction

Although breast cancer is becoming more curable, the cancer burden continues to increase as life expectancy rises [1]. It is estimated that 1 in 8 women will develop breast cancer during their lifetime [1] and despite early diagnostic approaches and numerous available drugs, breast cancer remains the leading cause of cancer deaths in women (626,679 deaths worldwide in 2018) [2]. In the vast majority of breast cancer patients, the cause of death is the development of resistance to therapy and the development of distant metastases [3–5].

Inflammation is a hallmark of cancer and plays an important role in tumor development and progression [6]; however, there are still many unknown players and mechanisms that regulate inflammatory processes in cancer. TNF-α/TNFR1/NF-κB is one of the major axis regulating inflammatory and immune processes in tumors [7]. TNF-α is a pro-inflammatory cytokine that is present in the tumor microenvironment. One of its main functions is to activate NF-κB signaling by binding to tumor necrosis factor receptor 1 (TNFR1) [7]. This regulatory axis can promote or suppress tumor progression, depending on the context. On the one hand, constitutive activation of NF-κB could lead to chronic inflammation and activation of pro-tumorigenic processes such as cell proliferation, survival, invasion, and angiogenesis [8, 9]. On the other hand, studies have also shown that NF-κB is required for the activation of the anti-tumor immune response; a disrupted activation of NF-κB signaling may help cancer cells escape from the host immune response [9–12]. As there are still many unanswered questions about the effect of the TNF-α/TNFR1/NF-κB signaling pathway in cancer, it is critically important to identify transcription factors involved in the regulation of this axis.

ETV7 is a transcription factor belonging to the large family of ETS (E26 Transforming Specific) transcription factors. It is a transcriptional repressor known to be up-regulated in many cancer types [13, 14]. For example, ETV7 was found to be up-regulated in 85% of medulloblastoma cases, and another study identified ETV7 as one of the 10 most up-regulated proteins in hepatocellular carcinoma [15]. In 2016, Piggin and colleagues reported an increased expression of ETV7 in all types of breast cancer compared to normal breast tissue. Interestingly, the expression of ETV7 correlated with the tumor’s aggressiveness [16]. Previous studies showed that ETV7 promotes tumor progression by acting on various molecular and cellular pathways [13, 17, 18]. Studies performed in our laboratory also demonstrated that different DNA-damaging agents up-regulated the expression of ETV7 in breast cancer cells. In the same study, we uncovered that breast cancer cells stably over-expressing ETV7 develop resistance to doxorubicin by repressing the *DNAJC15* gene, thereby increasing the expression of ABC pumps and leading to the increased efflux of the doxorubicin [19]. In addition, ETV7 modulates the plasticity of breast cancer stem cells, hence reducing the sensitivity of cancer cells to some anti-cancer drugs (e.g., doxorubicin, 5-fluorouracil) [18]. Another study demonstrating the pro-tumorigenic activities of ETV7 reported that ETV7 could form a complex with mTOR, called mTORC3, resulting in resistance to rapamycin (an mTOR inhibitor). In addition, genome-wide transcriptional profiling has demonstrated that the combined treatment of MCF7 breast cancer-derived cells with the chemotherapeutic agent doxorubicin and the inflammatory cytokine TNF-α results in the synergistic induction of ETV7 [20]. ETV7 was also identified as an interferon (IFN)-stimulated gene (ISG), and its expression is known to be up-regulated upon the treatment with type I, type II, and type III interferons [21–24]. We have recently shown that ETV7 negatively regulates the type I interferon response, and it is known to be involved in the viral immune response by suppressing a subset of ISGs that are important for the control of influenza and SARS-CoV-2 viruses [25]. However, the role of ETV7 in breast cancer immunity and inflammatory processes remains to be investigated. And, since immunotherapies appear to have great potential in the treatment of solid tumors [26, 27], a better understanding of the mechanisms regulating immune and inflammatory responses in breast cancer is essential for the successful development and application of novel therapeutic strategies.

In this study, we demonstrate that ETV7 plays a role in the inflammatory response in breast cancer-derived cell lines. We also show that ETV7 directly down-regulates the *TNFRSF1A* gene and reduces the activation of NF-κB signaling, thereby suppressing the inflammatory response. Moreover, we unveil a negative crosstalk between ETV7 and STAT3 in the regulation of the *TNFRSF1A* gene, and this crosstalk highlights the importance of ETV7 in cancer immunity and inflammation. Taken collectively, we suggest ETV7 as a novel regulator of *TNFRSF1A* gene expression and, therefore, a modulator of NF-κB signaling.

## Results

### ETV7 is involved in inflammatory and immune responses

**Fig. 1:**
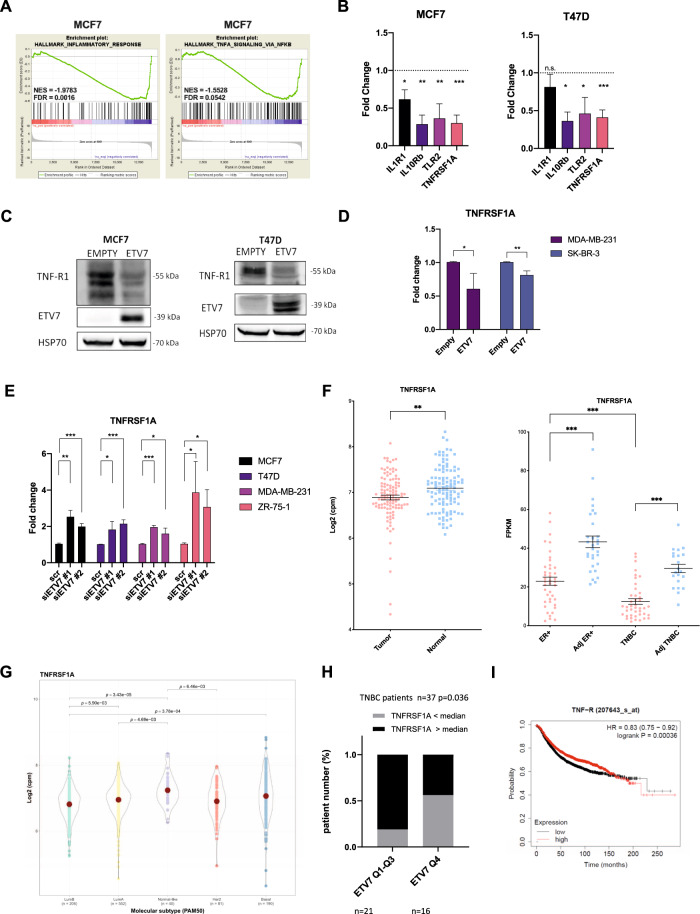
ETV7 modulates the inflammatory and immune responses in breast cancer. **A** Gene Set Enrichment Analysis of MCF7 cells over-expressing ETV7 or its Empty counterpart. Enrichment plot for the inflammatory response in MCF7 cells (on the left) and TNFA signaling via NF-κB in MCF7 cells (on the right) gene sets of the Hallmark Collection. The Normalized Enrichment Score (NES) shows the degree of the enrichment of the gene set; the negative sign indicates that the gene set is down-regulated in cells over-expressing ETV7. FDR = False Discovery Rate. **B** RT-qPCR analysis for the validation of genes involved in inflammation and immune response in MCF7 (on the left) and T47D (on the right) cells over-expressing ETV7 or Empty vector. Bars represent the averages and standard deviations of at least three biological replicates. **C** The expression of TNFRSF1A at the protein level in MCF7 and T47D cells over-expressing ETV7 or its empty counterpart. On the right of each blot is indicated the approximate observed molecular weight. HSP70 was used as a loading control. **D** RT-qPCR analysis of the normalized expression of the TNFRSF1A gene in MDA-MB-231 and SK-BR-3 cells transiently over-expressing ETV7 or harboring its Empty counterpart. Bars demonstrate the averages and standard deviations of at least three biological replicates. **E** RT-qPCR analysis of the normalized expression of the *TNFRSF1A* gene in MCF7, T47D, MDA-MB-231, and ZR-75-1 cells transfected with ETV7 targeting siRNA #1 and siRNA #2 or the scrambled control. **F** On the left, a dot plot demonstrating the differential expression analysis for the *TNFRSF1A* gene in a BRCA-matched patients’ dataset from TCGA (The Cancer Genome Atlas) database. Tumor (light red), normal (light blue). On the right, a dot plot of TNFRSF1A expression in ER-positive and triple-negative breast cancer patient cohort when comparing tumor tissue (light red) with matched adjacent tissue (light blue). ER+—Estrogen Receptor-positive; TNBC—triple-negative breast cancer; Adj—adjacent tissue. Indicated are the means and the SEMs. **G** TNFRSF1A expression in TCGA BRCA samples classified by PAM50 molecular subtypes. **H** Correlation analysis between ETV7 and TNFRSF1A mRNA level in TNBC patients from University Medical Center Hamburg-Eppendorf. Significance and numerosity are indicated. **I** Kaplan–Meier curves for RFS from a breast cancer cohort according to the relative expression of TNFRSF1A obtained with the KM plotter tool. The number of patients is shown below the graph. HR (Hazardous Ratio) and the statistical analyses are reported in the right corner of the graph. Whole panel: *p ≤ 0.05; **p ≤ 0.01; ***p ≤ 0.001; n.s. not significant.

To better understand the transcriptional networks regulated by ETV7, in our previous study, we performed transcriptome analyses in two breast cancer-derived cell lines, MCF7 and T47D, that stably over-express ETV7 or empty counterpart [18]. Interestingly, gene ontology analysis of commonly down-regulated DEGs identified innate immune and inflammatory responses as the most significant terms (see Supplementary Fig. 4A from our recent study [18]). Furthermore, gene set enrichment analysis (GSEA) highlighted “inflammatory response” and “TNFA_signaling_via_NF-κB” (Fig.1A and Supplementary Fig. 1A) particularly in MCF7 cells (a clear but not statistically significant trend was visible also for T47D cells), confirming the involvement of ETV7 in these processes. As a result of these analyses, we obtained a list of down-regulated genes known to be involved in inflammatory pathways. For further validation, we selected a set of genes with a Fold Change lower than -1.2 in both MCF7 and T47D cell lines. Using RT-qPCR, we demonstrated the repression of all selected targets (TNFRSF1A, IL10RB, IL1R1, and TLR-2) in MCF7 and T47D (Fig. 1B) cells, with the sole exception of IL1R1, which was significantly down-regulated only in MCF7 cells.

### ETV7 represses TNFRSF1A

We were particularly interested in the *TNFRSF1A* gene as it encodes for the TNFR1 receptor, which is the main receptor for TNF-α. We demonstrated that the expression of TNFRSF1A is significantly down-regulated in both MCF7 and T47D cells over-expressing ETV7. Furthermore, we were able to confirm the down-regulation of TNFRSF1A also in two other breast cancer-derived cell lines, SK-BR-3 and MDA-MB-231, upon the transient over-expression of ETV7 (Fig. 1D). To verify whether the repression of TNFRSF1A could be also observed upon the modulation of the endogenous level of ETV7, we treated MCF7, T47D, MDA-MB-231, and SK-BR-3 parental cell lines with DNA-damaging drugs (Doxorubicin and 5-FU), which are known from our previous publications [18, 19] to induce the expression of ETV7 (Supplementary Fig. 1E, F). Notably, after the treatment with Doxorubicin and 5-FU we observed a significant down-regulation of TNFRSF1A in MCF7, T47D, MDA-MB-231, and SK-BR-3 cells (Supplementary Fig. 1C, D). To understand whether this repression at the mRNA level was also reflected at the protein level, we performed Western blot analysis, demonstrating that ETV7 significantly down-regulated TNFR1 protein in both MCF7 and T47D cells (Fig. 1C). At the protein level, a slight down-regulation of TNFR1 was also observed in MDA-MB-231 cells, while in SK-BR-3 cells the difference was not visible (Supplementary Fig. 1B). Moreover, to determine whether the repression of TNFRSF1A was regulated by ETV7, we knocked-down ETV7 in MCF7, T47D, MDA-MB-231, and ZR-75-1 cells, using ETV7 targeting siRNA #1 and siRNA #2. The successful knock-down of ETV7 protein was validated by Western blot analysis (Supplementary Fig. 1G–J). The silencing of ETV7 was sufficient to revert the down-regulation of TNFRSF1A in MCF7, T47D, MDA-MB-231, and ZR-75-1 cells, strengthening the role of ETV7 in the regulation of TNFRSF1A in various cellular models (Fig. 1E).

It is known from the literature that the increased expression of ETV7 has also been detected in breast cancer patients and it correlated with breast cancer aggressiveness [16]. Thus, we were intrigued to understand if ETV7 affects the expression of TNFRSF1A also in breast cancer patients. Firstly, we analyzed the expression of ETV7 in breast cancer patients compared to normal breast tissue, using samples from TCGA and a private cohort, including ER-positive and triple-negative breast cancer patients. In both cohorts, we showed an increase in ETV7 levels in breast cancer tissues compared to normal tissues (Supplementary Fig. 1K). Subsequently, we also analyzed the expression of TNFRSF1A in breast cancer patients compared to normal breast tissue. We performed gene expression analysis using the TCGA database and observed a decrease in TNFRSF1A levels in breast cancer tissues (BRCA dataset) compared to matched normal tissues (Fig. 1F). Furthermore, we expanded our analysis and studied the expression of TNFRSF1A in an additional private cohort of breast cancer patients. Also in this patient cohort, we observed a significant decrease in TNFRSF1A in both ER-positive and triple-negative breast cancer tissues. Furthermore, the expression levels of TNFRSF1A in more aggressive triple-negative tumors were lower than in ER-positive tumors, suggesting that TNFRSF1A could have a potential prognostic value (Fig. 1F). Furthermore, the expression of TNFRSF1A was significantly lower in all molecular types of breast cancer compared to normal tissue (Fig. 1G). Additionally, some of the other genes repressed by ETV7 (TLR2 and IL1R1) were also lower in BC in comparison with normal tissues (Supplementary Fig. 1L). Eventually, we also tried to correlate the high expression of ETV7 with the lower levels of TNFRSF1A and vice versa. Although this correlation was not significant in the whole cohort (Supplementary Fig. 1N), we observed an inverse correlation between ETV7 and TNFRSFIA expression in the TNBC subgroup. Specifically, BC patients with higher ETV7 levels were more frequent in the group of BC patients with lower TNFRSF1A levels (below the median) (Fig. 1H). This observation is in line with our previously published data showing that the highest ETV7 levels are found in TNBC [18].

Afterwards, we investigated whether the expression level of TNFRSF1A influences the survival of breast cancer patients. Using the Kaplan–Meier plotter tool (TCGA dataset), we analyzed the impact of TNFRSF1A expression in breast cancer patients and confirmed a significant correlation between lower TNFRSF1A levels and poor prognosis of breast cancer patients (Fig. 1I). The same result was also confirmed analyzing the survival data from another private cohort (Supplementary Fig. 1M).

### ETV7 directly down-regulates TNFRSF1A

**Fig. 2:**
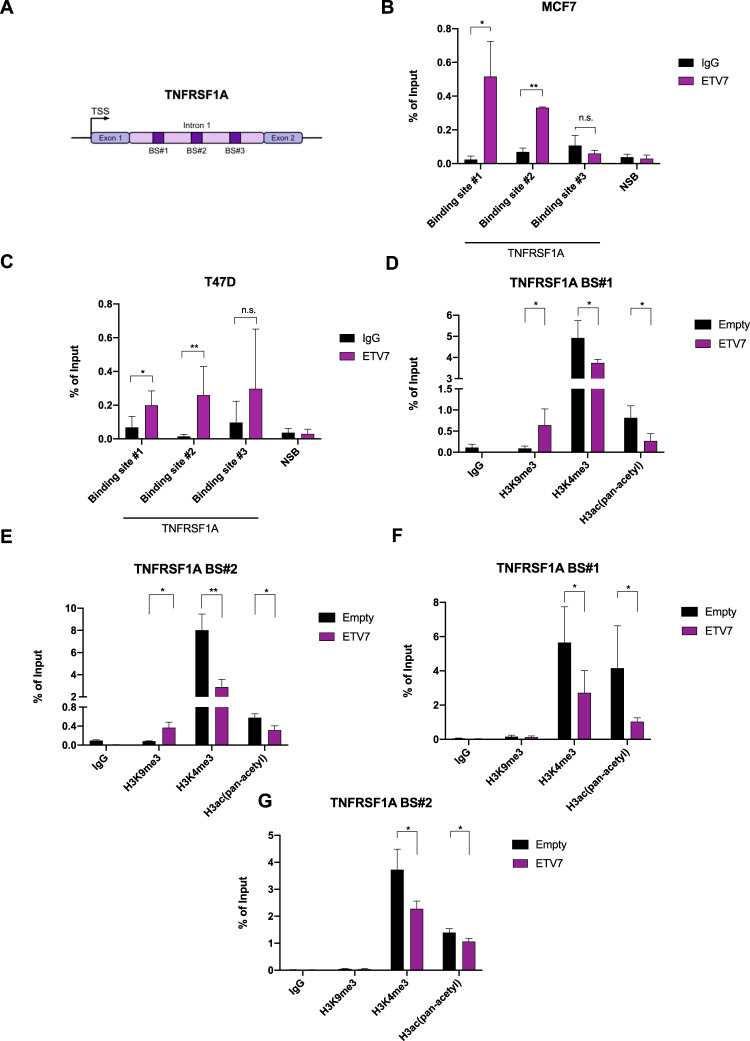
ETV7 directly represses the expression of TNFRSF1A in breast cancer cells. **A** A schematic view of the TNFRSF1A Intron 1 and the studied ETV7 binding sites. TNFRSF1A BS#1 is located +5483 bp from the Transcription Start Site (TSS); BS#2 + 5627 bp from TSS; BS#3 + 6069 bp from TSS. **B**, **C** ChIP-qPCR of TNSFRSF1A Intron 1 in MCF7 (**B**) or T47D (**C**) cells over-expressing ETV7. The percentage of the enrichment of ETV7 or control (normal mouse IgG) bound to TNFRSF1A Intron 1 with respect to input DNA is shown. NSB—non-specific binding, the *ACTB* promoter. **D**, **E** Chip-qPCR assessing H3K9me3, H3K4me3, and H3ac (pan-acetyl) deposition at the TNFRSF1A Intron 1 binding site #1 (**D**, **F**) and binding site #2 (**E**, **G**) in MCF7 and T47D over-expressing ETV7 or its empty counterpart. The percentage of the enrichment of ETV7 or control (normal rabbit IgG) bound to TNFRSF1A Intron 1 with respect to input DNA is shown. Whole panel: Bars represent the averages and standard deviations of at least three biological replicates. *p ≤ 0.05; **p ≤ 0.01; ***p ≤ 0.001, n.s. not significant.

Given that ETV7 is reported to be a transcriptional repressor, to understand whether it could directly regulate the TNFRSF1A expression, we searched for putative ETV7 binding sites in the regulatory elements of the *TNFRSF1A* gene. Based on ETV7 consensus sequences [28–30], we identified three potential binding sites for ETV7 containing the GGAA motif in the first intron of TNFRSF1A (Fig. 2A). To understand whether the transcription repression was associated with the direct binding of ETV7 to these regulatory elements in the *TNFRSF1A* gene, we performed chromatin immunoprecipitation followed by qPCR. We were able to demonstrate the direct binding of ETV7 to the *TNFRSF1A* intron regions (BS#1 and BS#2) in both MCF7 (Fig. 2B) and T47D (Fig. 2C) cells. Furthermore, to gain insights into the mechanism underlying the ETV7-mediated transcriptional repression of TNFRSF1A, we analyzed the activating and repressive histone marks on *TNFRSF1A* regulatory regions (binding site #1 and #2). Using chromatin immunoprecipitation, we were able to demonstrate the decrease in tri-methylation of lysine 4 on histone H3 (H3K4me3) and in acetylation on several lysine residues on H3 (H3K9ac, H3K14ac, H3K18ac, H3K23ac, H3K27ac), well-established markers of open chromatin, in both MCF7 and T47D cells and an increase in tri-methylation of lysine 9 on histone H3 (H3K9me3), instead a marker of closed chromatin, in MCF7 cells, hence confirming that ETV7 can directly repress TNFRSF1A by reducing the accessibility of chromatin (Fig. 2D–G).

### ETV7 reduces NF-κB activation by repressing TNFRSF1A

**Fig. 3:**
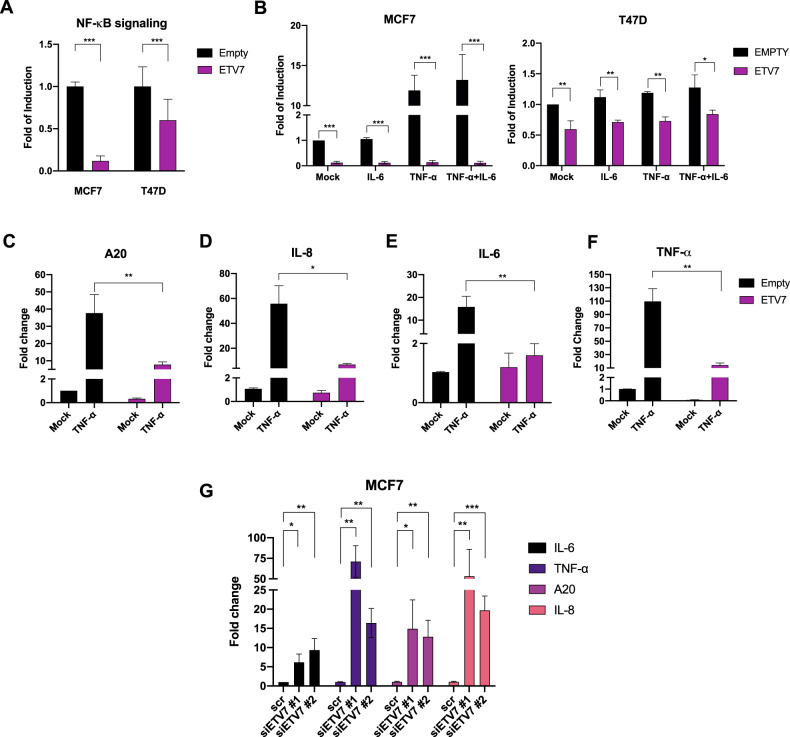
ETV7 reduces NF-κB signaling by repressing TNFRSF1A. **A** Gene reporter assays in MCF7 and T47D cells over-expressing ETV7 or its empty counterpart transiently transfected with the pGL3-NF-κB reporter plasmid. Data is normalized using the *Renilla reniformis* luciferase reporter vector pRL-SV40 and shown as fold of induction relative to the Empty control. **B** Gene reporter assays in MCF7 (on the left) and T47D (on the right) cells over-expressing ETV7 or its empty counterpart transfected with the pGL3-NF-κB reporter plasmid and stimulated with TNF-α (10 ng/ml for MCF7 and 15 ng/ml for T47D), IL-6 (20 ng/ml) or combination of both for 4 h. Data are normalized using the *Renilla reniformis* luciferase reporter vector pRL-SV40 and shown as fold of induction relative to the Empty control. **C**–**F** RT-qPCR analysis of known NF-κB target genes: A20 (**C**), IL-8 (**D**), IL-6 (**E**), and TNF-α (**F**) in MCF7 ETV7 or Empty cells treated with TNF-α (10 ng/ml) for 4 h. Bars represent the averages and standard deviations of at least three biological replicates. **G** RT-qPCR analysis of the normalized expression of NF-κB target genes in MCF7 parental cells transfected with ETV7 targeting siRNA #1 and siRNA #2 or the scrambled control. Whole panel: Bars represent the averages and standard deviations of at least three biological replicates. *p ≤ 0.05; **p ≤ 0.01; ***p ≤ 0.001, n.s. not significant.

**Fig. 4:**
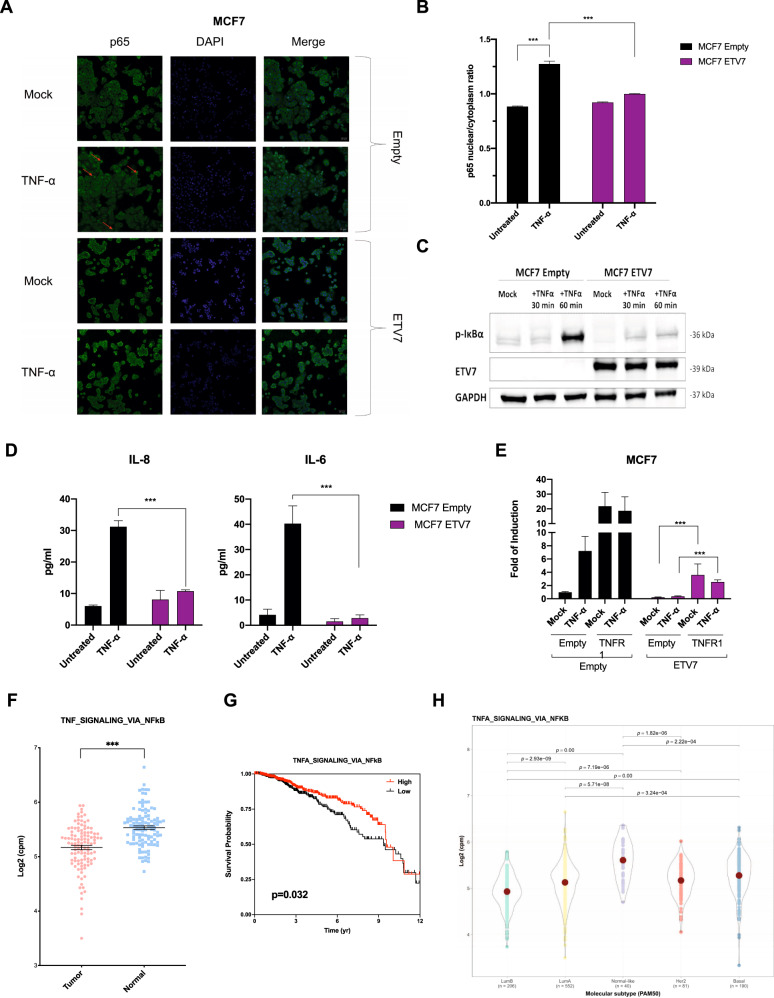
ETV7 suppresses NF-κB activation and inflammatory response. **A** Immunofluorescence analysis for the p65 (green signal) nuclear translocation in MCF7 Empty and MCF7 ETV7 cells. Nuclei are stained in blue. MCF7 cells were left untreated or stimulated with 10 ng/ml TNF-α for 60 min. Shown are representative data for at least three biological replicates. Images were acquired with a ×20 magnification. The arrows indicate the nuclear localization of p65. **B** Quantification of nuclear:cytoplasmic ratios of p65 fluorescence intensity in MCF7 Empty and MCF7 ETV7 cells. Bars represent mean ± standard deviation from analysis of 10 (per each biological replicate; *n* = 3) separated field images. **C** Western blot analysis of phosphorylated IκBα in MCF7 Empty and ETV7 cells in response to 10 ng/ml TNF-α treatment for 1 h. On the right of each blot is indicated the approximate observed molecular weight. GAPDH was used as a loading control. **D** The secretion of IL-8 and IL-6 in the supernatant of MCF-7 Empty and MCF7 ETV7 cells without or with stimulation with TNF-α (10 ng/ml), measured by ELISA. Bars represent the mean and the standard deviation of three biological replicates. **E** Gene reporter assays in MCF7 Empty or MCF7 ETV7 cells transfected with the pGL3-NF-κB reporter vector and pcDNA3.1-TNFR1 or pcDNA3.1-Empty plasmids and untreated or treated with 10 ng/ml TNF-α for 4 h. Data is normalized using the *Renilla reniformis* luciferase reporter vector pRL-SV40 and shown as fold of induction relative to the Empty untreated control. **F** A dot plot demonstrating the differential expression analysis for the TNF_SIGNALING_VIA_NFkB gene set in a BRCA-matched patients’ dataset from TCGA (The Cancer Genome Atlas) database. Tumor (light red), Normal (light blue). **G** Kaplan–Meier curves for TCGA breast cancer patients stratified according to the average expression of TNFA_SIGNALING_VIA_NFkB gene signature. Curves represent the probability of disease-specific survival (DSS). *p*-values are calculated with the log-rank test. **H** TNFA_SIGNALING_VIA_NFkB gene set expression in TCGA BRCA samples classified by PAM50 molecular subtypes. *p*-values are shown when significant. Whole panel: *p ≤ 0.05; **p ≤ 0.01; ***p ≤ 0.001.

Knowing that TNF-α activates NF-κB signaling by binding to the TNFR1 receptor, we hypothesized that the ETV7-mediated repression of TNFRSF1A modulates the transcriptional activity of NF-κB in MCF7 and T47D breast cancer cells. Firstly, we performed a gene reporter assay using the pGL3-NF-κB reporter plasmid (Supplementary Fig. 2A) in MCF7 and T47D cells over-expressing ETV7 or Empty vector. Noteworthy, the over-expression of ETV7 resulted in significant repression of the basal NF-κB transcriptional activity in both MCF7 and T47D (Fig. 3A) cells. To understand whether this phenomenon can also be observed upon NF-κB induction, we repeated the experiment stimulating the cells with TNF-α (the main NF-κB activator), IL-6 (a broader pro-inflammatory cytokine), or a combination of these two cytokines to stimulate the transcriptional activity further. Notably, we demonstrated that ETV7-over-expressing cells did not induce the NF-κB signaling even after the stimulation (Fig. 3B). This effect was particularly strong in MCF7 cells, whereas T47D cells were overall less responsive to TNF-α stimulation, due to the already high endogenous levels of NF-κB signaling. Next, we investigated whether the ETV7-mediated reduction in NF-κB activation affects NF-κB target genes’ expression. We measured the mRNA expression of 4 well-known NF-κB-regulated targets, such as TNF-α, IL-8, IL-6, and A20 [31, 32]. The over-expression of ETV7 significantly reduced the TNF-α-dependent expression of all of these genes in both MCF7 (Fig. 3C–F) and T47D (Supplementary Fig. 2B–E) cells, with the sole exception of IL-8, which expression was significantly reduced only in MCF7 cells. Furthermore, to confirm if the expression of these NF-κB targets can be bi-directionally mediated by ETV7, we knock-down ETV7 using siRNAs in MCF7 parental cells and analyzed the expression of IL-6, IL-8, TNF-α, and A20 genes. Importantly, upon the silencing of ETV7, we demonstrated a significant up-regulation of all four genes, which supports the role of ETV7 in the regulation of NF-κB signaling (Fig. 3G and Supplementary Fig. 1G). To make the result even more significant, we expanded the panel of cell lines and performed the same analysis in T47D, MDA-MB-231, and ZR-75-1 cells, confirming also in these additional models the role of ETV7 in the regulation of NF-κB pathway (Supplementary Fig. 1H–J and Supplementary Fig. 2F–H). Moreover, the activation and translocation of NF-κB into the nucleus are initiated by the phosphorylation, ubiquitination, and proteolytic degradation of IκBα by the IKK complex [33, 34]. Therefore, we decided to investigate the effect of ETV7 over-expression on the phosphorylation of IκBα. Interestingly, the over-expression of ETV7 led to a significant reduction in phosphorylated IκBα in both MCF7 and T47D cell lines (Fig. 4C and Supplementary Fig. 2I). Furthermore, to understand if the reduction of phosphorylated IκBα affects the nuclear localization of p65, we performed immunofluorescence analysis on MCF7 and T47D cells harboring an Empty vector or over-expressing ETV7 and we showed that in both MCF7 and T47D cells there was a significant decrease in the translocation of p65 to the nucleus upon the stimulation of NF-κB pathway with TNF-α (Fig. 4A, B and Supplementary Fig. 2J, K). Furthermore, to confirm if the repression of NF-κB signaling results in the reduced secretion of pro-inflammatory factors, hence reducing the inflammatory response, we determined the levels of IL-8, IL-6, and TNF-α in MCF7 and T47D cell culture supernatants by enzyme-linked immunosorbent assay (ELISA). Notably, after the stimulation with TNF-α, we observed the significant decrease of IL-6 and IL-8 in the medium from ETV7-over-expressing cells, both in MCF7 and T47D (Fig. 4D and Supplementary Fig. 3F). Moreover, even though the basal level of TNF-α was very low, we were able to demonstrate the significant decrease in secreted TNF-α both in MCF7 ETV7 and T47D ETV7 cell supernatants (Supplementary Fig. 3E). To confirm the hypothesis that the repression of NF-κB signaling depends, at least partially, on the ETV7-mediated repression of TNFRSF1A, we over-expressed TNFR1 in MCF7 ETV7 and MCF7 Empty cells and performed a gene reporter assay using the pGL3-NF-κB reporter plasmid (Supplementary Fig. 3A). The over-expression of TNFR1 was verified in all the conditions by Western blot analysis in both MCF7 Empty and MCF7 ETV7 cells (Supplementary Fig. 3B). Notably, upon the over-expression of TNFR1, we detected a partial, but significant, increase in the transcriptional activity of NF-κB in MCF7 cells over-expressing ETV7 (Fig. 4E). A similar trend was also seen by analyzing NF-κB targets’ expression IL-8 and IL-6, upon the rescue with TNFR1-encoding plasmid (Supplementary Fig. 3C, D). Besides, to give a more translational significance to our results, we decided to compare the expression of TNF_SIGNALING_VIA_NFkB gene set in breast cancer tissues versus normal tissues. Indeed, using the TCGA database, we observed a decrease in the expression of TNF_SIGNALING_VIA_NFkB signature genes in breast cancer tumor tissues (BRCA dataset) in comparison with normal tissues (Fig. 4F). Besides, the significant reduction in several NF-κB target genes in breast cancer patient tissues was also observed by analyzing them separately (Supplementary Fig. 3G). Furthermore, the repression of TNF_SIGNALING_VIA_NFkB, as well as INFLAMMATORY_RESPONSE signature genes was also confirmed when analyzing the different molecular subtypes of breast cancer (Fig. 4H and Supplementary Fig. 3I). To understand whether the repression of NF-κB pathway could have a prognostic value for breast cancer patients, we performed survival analysis on TCGA database samples and confirmed a significant correlation between lower NF-κB and inflammatory response gene signature levels and a poorer prognosis of breast cancer patients (Fig. 4G and Supplementary Fig. 3H).

### ETV7 competes with STAT3 in the regulation of the *TNFRSF1A* gene

**Fig. 5:**
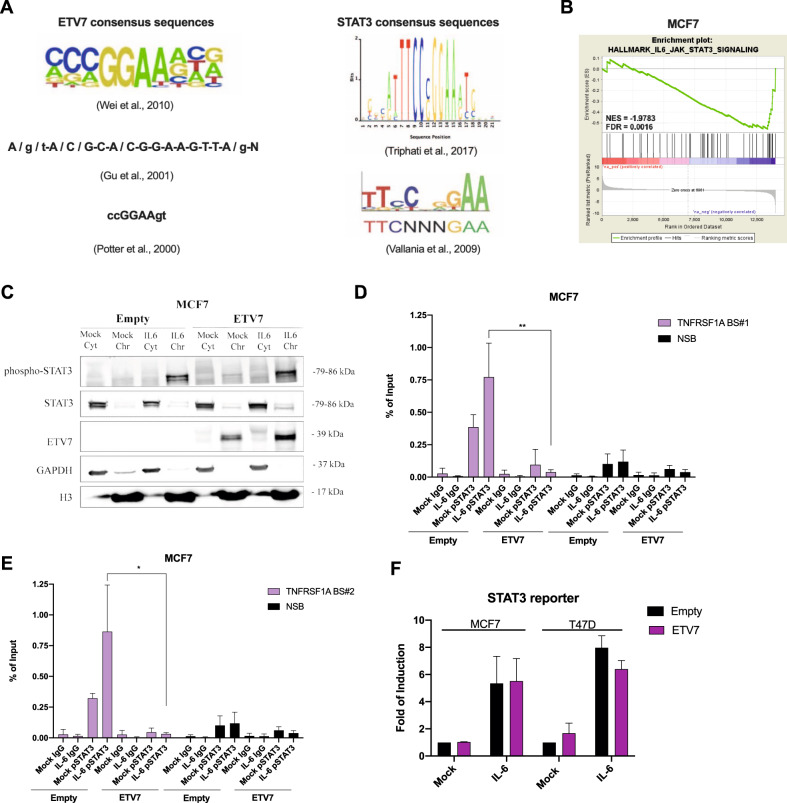
Crosstalk between ETV7 and STAT3. **A** Canonical ETV7 and STAT3 binding sites known from the literature. **B** Gene Set Enrichment Analysis of MCF7 cells over-expressing ETV7 or its Empty counterpart. Enrichment plot for IL6_JAK_STAT3 signaling in MCF7 cells gene sets of the Hallmark Collection. The Normalized Enrichment Score (NES) shows the degree of the enrichment of the gene set; the negative sign indicates that the gene set is down-regulated in cells over-expressing ETV7. FDR = False Discovery Rate. **C** Western blot analysis of subcellular fractionation from MCF7 Empty and MCF7 ETV7 cells untreated or treated with IL-6 (20 ng/ml) for 4 h. On the right of each blot is indicated the approximate observed molecular weight. Cyt—cytoplasmic protein fraction, Chr—chromatin-enriched protein fraction. GAPDH was used as a loading control for the cytoplasmic fraction. Histone H3 was used as a loading control for chromatin-enriched protein fraction. **D**, **E** ChIP-qPCR of TNSFRSF1A Intron 1 Binding site #1 (**D**) and Binding site #2 (**E**) in MCF7 cells over-expressing ETV7 untreated or treated with IL-6 (20 ng/ml) for 4 h. The percentage of the enrichment of pSTAT3 or control (normal rabbit IgG) bound to TNFRSF1A Intron 1 with respect to Input DNA is shown. NSB—non-specific binding, a region within the *ACTB* promoter. BS —binding site. Bars represent the averages and standard deviations of at least three biological replicates. F) Gene reporter assays in MCF7 Empty/ETV7 (on the left) and T47D Empty/ETV7 (on the right) cells transfected with M67-STAT3 reporter untreated or treated with IL-6 (20 ng/ml) for 4 h. Data are normalized using the *Renilla reniformis* luciferase reporter vector pRL-SV40 and shown as fold of induction relative to the Empty untreated control. Whole panel: *p ≤ 0.05; **p ≤ 0.01; ***p ≤ 0.001.

It is known from the literature that STAT3 can up-regulate the expression of TNFRSF1A by binding to its first intron, the same intron bound by ETV7 for the regulation of TNFRSF1A [31]. Moreover, there are similarities between the binding sites for STAT3 and ETV7 (Fig. 5A) [35, 36]. Hence, we investigated the potential crosstalk between these two transcription factors. Indeed, one of the binding regions (ETV7 BS #2) we identified also contains a binding site for STAT3. The potential interaction between STAT3 and ETV7 was also supported by our RNA-seq analysis, as it demonstrated a down-regulation of IL-6_JAK_STAT3 signaling in cells over-expressing ETV7; this was particularly evident for MCF7 cells (Fig. 5B), but a trend (even if not statistically significant) was visible also for T47D cells (Supplementary Fig. 4A).

In normal conditions, STAT3 can be activated by external stimuli such as interferons, interleukins, and TNF-α. IL-6 is known to be one of the most potent activators of STAT3; therefore, we chose to use the stimulation with IL-6 to activate STAT3. To test whether IL-6 can also trigger the phosphorylation of STAT3 in our cellular model and to validate the subcellular localization of ETV7 and STAT3 in endogenous conditions and after the treatment with IL-6, we performed the subcellular protein fractionation followed by Western blot analysis. Data obtained confirmed that the phosphorylated form of STAT3 is mainly located in the chromatin-associated fraction and, as expected, there is an increase in phosphorylated STAT3 in the chromatin compartment in response to the treatment with IL-6 (Fig. 5C and Supplementary Fig. 4B, D). ETV7 protein is located in both the cytoplasmic fraction and the chromatin-associated fraction and does not appear to be affected by the treatment with IL-6/activation of STAT3. We then analyzed the effects of STAT3 activation on the expression of the *TNFRSF1A* gene in MCF7 cells over-expressing ETV7 or an empty counterpart. Using RT-qPCR, we found that although the treatment with IL-6 induced TNFRSF1A in MCF7 empty cells, there was a significant reduction in the expression of TNFRSF1A in the cells over-expressing ETV7 (Supplementary Fig. 4C). These results confirm that ETV7 can down-regulate TNFRSF1A despite STAT3 activation.

As mentioned previously, the ETV7 binding region in TNFRSF1A is located in the first intron, and it is known from the literature that STAT3 can also bind the same region [31]. Therefore, we performed a chromatin immunoprecipitation assay to understand whether ETV7 could compete with STAT3 for binding TNFRSF1A intron 1. We treated ETV7 over-expressing cells with IL-6, which activates STAT3, and performed immunoprecipitation using an antibody for the phosphorylated version of STAT3. The activation of STAT3 was confirmed by performing Western blot analysis for phosphorylated STAT3, and ETV7 over-expression did not alter it (Supplementary Fig. 4E). We then analyzed by qPCR the three regions containing binding sites for ETV7 as well as STAT3. Our results demonstrated that upon the over-expression of ETV7, the ability of STAT3 to bind the 1st and the 2nd regulatory regions in TNFRSF1A was remarkably decreased in MCF7 cells. The tendency of the reduction in STAT3 binding to the 3rd regulatory region was also visible but not statistically significant (Fig. 5D, E and Supplementary Fig. 4G).

To better understand the crosstalk between these two transcription factors in regulating TNFRSF1A expression, we tried to determine whether ETV7 could affect the STAT3 signaling. Firstly, to verify if the over-expression of ETV7 impacted the mRNA expression of STAT3, we analyzed STAT3 expression in MCF7 and T47D cells over-expressing ETV7 and observed that upon the over-expression of ETV7 in both cell lines, especially in MCF7, STAT3 expression was significantly down-regulated (Supplementary Fig. 4F). However, this effect was not confirmed at the protein level (Fig. 5C, Supplementary Fig. 4D, E). Alternatively, to evaluate the impact of ETV7 on the transcriptional activity of STAT3, we performed gene reporter assays using a luciferase reporter construct containing four canonical STAT3 binding sites (4xM67 pTATA TK-Luc). Our results demonstrated that ETV7 did not affect the overall transcriptional activity of STAT3 (Fig. 5F). To further confirm that the displacement of STAT3 was specific for TNFRSF1A regulatory region and not to STAT3 targets in general, we performed chromatin immunoprecipitation with pSTAT3 and studied another known transcriptional target of STAT3, MED16 [36]. Notably, we did not observe a statistically significant difference between cells over-expressing ETV7 or empty vector, which confirms that the ETV7-mediated displacement of STAT3 is specific for the TNFRSF1A regulatory region (Supplementary Fig. 4H). Moreover, to understand if the endogenous modulation of ETV7 with DNA-damaging drugs is sufficient to disrupt STAT3 binding from the regulatory region of TNFRSF1A, we performed chromatin immunoprecipitation assay on the cells treated with IL-6 (activates STAT3) and Doxorubicin (induces ETV7). Interestingly, we confirmed that already the modulation of endogenous ETV7 levels with DNA-damaging agent was sufficient for the STAT3 displacement at the binding site #2 in TNFRSF1A Intron I in both MCF7 cells, and in both binding sites #1 and #2 in T47D cells (Supplementary Fig. 4I, J). Furthermore, we sought to understand whether ETV7 directly interacts with the STAT3 protein. To evaluate this putative interaction, we performed a co-immunoprecipitation (Co-IP) experiment, using antibodies against ETV7 for immunoprecipitation and detecting phosphorylated STAT3 in Western blot. According to the Co-IP results, we could not observe a direct interaction between ETV7 and STAT3 either in MCF7 or in T47D cells (Supplementary Fig. 4K). Overall, with this study we demonstrated the ETV7-mediated repression of TNFR1/NF-κB axis and uncovered the mechanism behind this effect depending on the competition with STAT3 in the transcriptional regulation of the TNFRSF1A gene.

### ETV7 protein level inversely correlated with TNFR1 in breast cancer patients

**Fig. 6:**
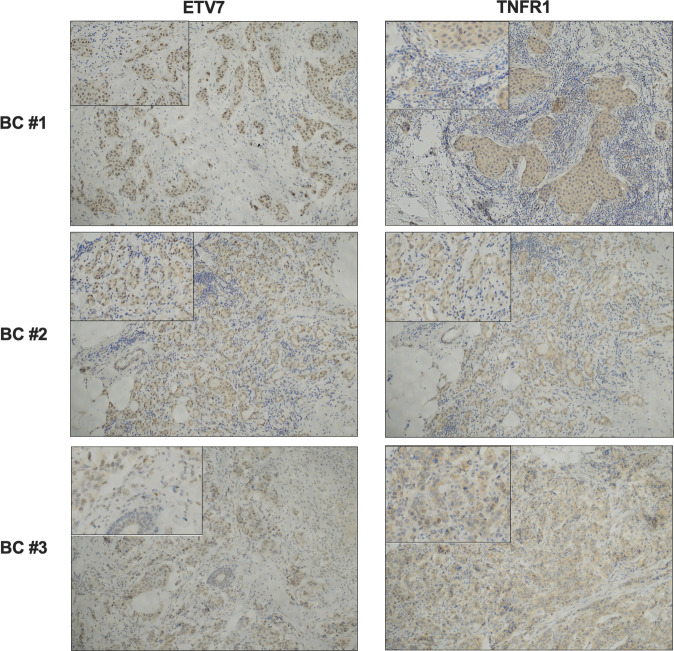
An inverse correlation between ETV7 and TNFR1 in BC patients. Three samples from BC patients with invasive ductal carcinoma were analyzed by IHC. Tissues were stained with antibodies against ETV7 and TNFR1 proteins and studied at ×10 magnification. A ×20 magnification insert is shown for each image. Top panels: tissues from a HER2-positive BC; middle panel: tissues from a triple-negative BC; bottom panels: tissues from a luminal A BC.

In order to verify whether an inverse correlation between ETV7 and TNFR1 is also evident at the protein level in breast cancer patients, IHC analyses were conducted in a limited number of samples (3 patients) with invasive ductal carcinoma of the breast. Interestingly, results confirmed that BC samples with higher levels of ETV7 presented a lower expression of TNFR1 (Fig. 6, top and middle panels). ETV7 signal was mainly nuclear, while TNFR1 was cytoplasmic, as expected. Conversely, a sample with lower levels of ETV7 showed a higher expression of TNFR1 (Fig. 6, bottom panels), further confirming the inverse correlation between these two proteins.

## Discussion

ETV7 is a transcriptional repressor that belongs to the family of ETS transcription factors [13]. It is known to be up-regulated in many cancer types [13–15]. In addition, Piggin and colleagues demonstrated that ETV7 expression was significantly higher in breast cancer tissues compared to normal breast tissue, suggesting that ETV7 may play an important role in breast cancer development and progression [16]. Our previous studies and those of other scientists have already shown that ETV7 is involved in the development of drug resistance to various DNA-damaging drugs as well as mTOR inhibitor rapamycin [17–19]. Furthermore, ETV7 is also well-known as an interferon (IFN)-stimulated gene. Interestingly, we recently demonstrated that ETV7 repressed several IFN-responsive genes, increasing the subpopulation of breast cancer stem-like cells and, thus, the resistance to chemo- and radiotherapy [18, 22–24].

Pieces of evidence from computational studies in cancer, as well as the already-known role of ETV7 in viral infections, indicate a potential function for ETV7 in cancer immunity and inflammation. However, the role of ETV7 in solid cancer tumor microenvironment, inflammation, and immune response remains to be studied [25, 37]. Therefore, in this study, we focused on deciphering the role of ETV7 in breast cancer immunity and inflammatory response. The RNA-seq analysis we had previously conducted on the breast cancer-derived cells MCF7 and T47D over-expressing ETV7 or its empty counterpart supported our hypothesis by demonstrating the involvement of ETV7 in inflammation and immune responses (Fig. 1A and Supplementary Fig. 1A). We validated several putative targets—TLR2, TNFRSF1A, IL1R1, IL10RB—that are down-regulated by ETV7 and known to be involved in inflammatory and immune processes and focused on a more in-depth analysis of the regulation of *TNFRSF1A* gene expression [38–40] (Fig. 1B).

In this study, we confirmed that the *TNFRSF1A* gene was significantly down-regulated by ETV7 at mRNA and protein levels both in MCF7 and T47D cell lines (Fig. 1C). We also observed the transcriptional repression of the *TNFRSF1A* gene in other breast cancer cell lines SK-BR-3 and MDA-MB-231 upon transient over-expression of ETV7 (Fig. 1D). Furthermore, we demonstrated that the knock-down of ETV7 restored the expression of TNFRSF1A in several breast cancer-derived cellular models (Fig. 1E).

Interestingly, in a private cohort of breast cancer patients, with triple-negative breast cancer patients’ subgroup we demonstrated an inverse correlation between the expression of ETV7 and TNFRSF1A (Fig. 1H). Moreover, we verified lower TNFRSF1A expression levels in breast cancer patients compared to normal breast tissues (from TCGA and in another private cohort, Fig. 1F) and this reduced TNFRSF1A correlated with a worse prognosis (Fig. 1I), which demonstrate the potential translational relevance of our observations. Moreover, in a limited number of local BC patients, ETV7 and TNFR1 protein levels were inversely correlated (Fig. 6).

We confirmed that the TNFRSF1A repression involves the direct binding of ETV7 to the Intron 1 of TNFRSF1A, and we identified three ETV7 binding sites in this region (Fig. 2A–C). Furthermore, we showed that ETV7 not only bind to the regulatory region of TNFRSF1A, but recruits chromatin remodelers responsible for a “closed” or “more condensed” conformation (i.e., HDACs and histone methyl-transferases) (Fig. 2D–G). This observation is consistent with the unique piece of information about ETV7 and chromatin remodelers, published by Boccuni and co-workers [41]. They showed that ETV7 can interact with H-L(3)MBT, a component of the Polycomb repressive complex.

*TNFRSF1A* encodes for Tumor Necrosis Factor Receptor 1 (TNFR1), one of the most critical transmembrane receptors for TNF-α. By binding to the TNFR1 receptor, TNF-α activates NF-κB signaling, a group of transcription factors including RelA/p65, RelB, c-Rel, p50, and p52 [42–44]. NF-κB is involved in the regulation of several critical cellular processes, such as proliferation, cell death, survival, and cellular homeostasis [44]. Furthermore, an essential function of NF-κB is the control of the immune response. Indeed, NF-κB regulates the expression of different genes involved in both innate and adaptive immune responses, as well as inflammation [45, 46].

Interestingly, STAT3, another master regulator of inflammation and immunity, is able to induce NF-κB activation by up-regulating TNFRSF1A, specifically by directly binding to its first intron, the same regulatory region also bound by ETV7 [31]. However, in the breast cancer cells over-expressing ETV7, we could not observe this regulatory mechanism, even when STAT3 was activated by the treatment with IL-6 (Supplementary Fig. 4C), thus demonstrating that there is a putative competitive relationship between ETV7 and STAT3 in the regulation of the TNFRSF1A gene. Given that the consensus motifs of STAT3 and ETV7 are similar (i.e., TTCCCGGAA and CA/CGGAAGT, respectively [28–30, 35, 36]), we searched for possible binding sites for ETV7 in the first intron of the *TNFRSF1A* gene that could also be used by STAT3 (Fig. 5A). Through chromatin immunoprecipitation, we demonstrated that ETV7 is able to reduce the binding of STAT3 to the Intron 1 of TNFRSF1A, confirming our hypothesis that ETV7 competes with STAT3 when the binding sites are close to each other (Fig. 5D, E and Supplementary Fig. 4D, I, J). To confirm further the competition between ETV7 and STAT3 in the regulation of *TNFRSF1A* gene, a reporter assay could be performed in cancer cells over-expressing ETV7 using a reporter vector containing the first intron of the *TNFRSF1A* gene.

According to the literature, TNFR1 is crucial for the activation of the NF-κB signaling pathway; therefore, we aimed to determine whether the ETV7-mediated repression of the *TNFRSF1A* gene also affects the activation of NF-κB. We confirmed that in breast cancer cells over-expressing ETV7, the repression of TNFRSF1A decreased the activation of NF-κB both in the basal state and upon the stimulation with TNF-α (Fig. 3A–D). The ETV7-mediated reduction in NF-κB signaling was more pronounced in MCF7 cells compared with T47D cells, as T47D cells were globally less responsive to TNF-α. We hypothesize that this reduced sensitivity to TNF-α is due to NF-κB activity in T47D cells, which is already high before the stimulation. The reduced activation of NF-κB was also confirmed by the detection of the reduced level of IκBα phosphorylation (Fig. 4C and Supplementary Fig. 2I) and the subsequent diminished nuclear accumulation of p65 (Fig. 4A, B and Supplementary Fig. 2J, K) in cells over-expressing ETV7.

Furthermore, we showed that this decreased NF-κB activation leads to a reduced expression of NF-κB target genes IL-8, IL-6, A20, and TNF-α, which are well-known to be involved in the inflammatory processes, indicating reduced inflammatory and immune processes (Fig. 3C–F and Supplementary Fig. 2B–E). Additionally, the silencing of ETV7 was able to restore the activation of these NF-κB targets (Fig. 3G and Supplementary Fig. 2F–H).

**Fig. 7:**
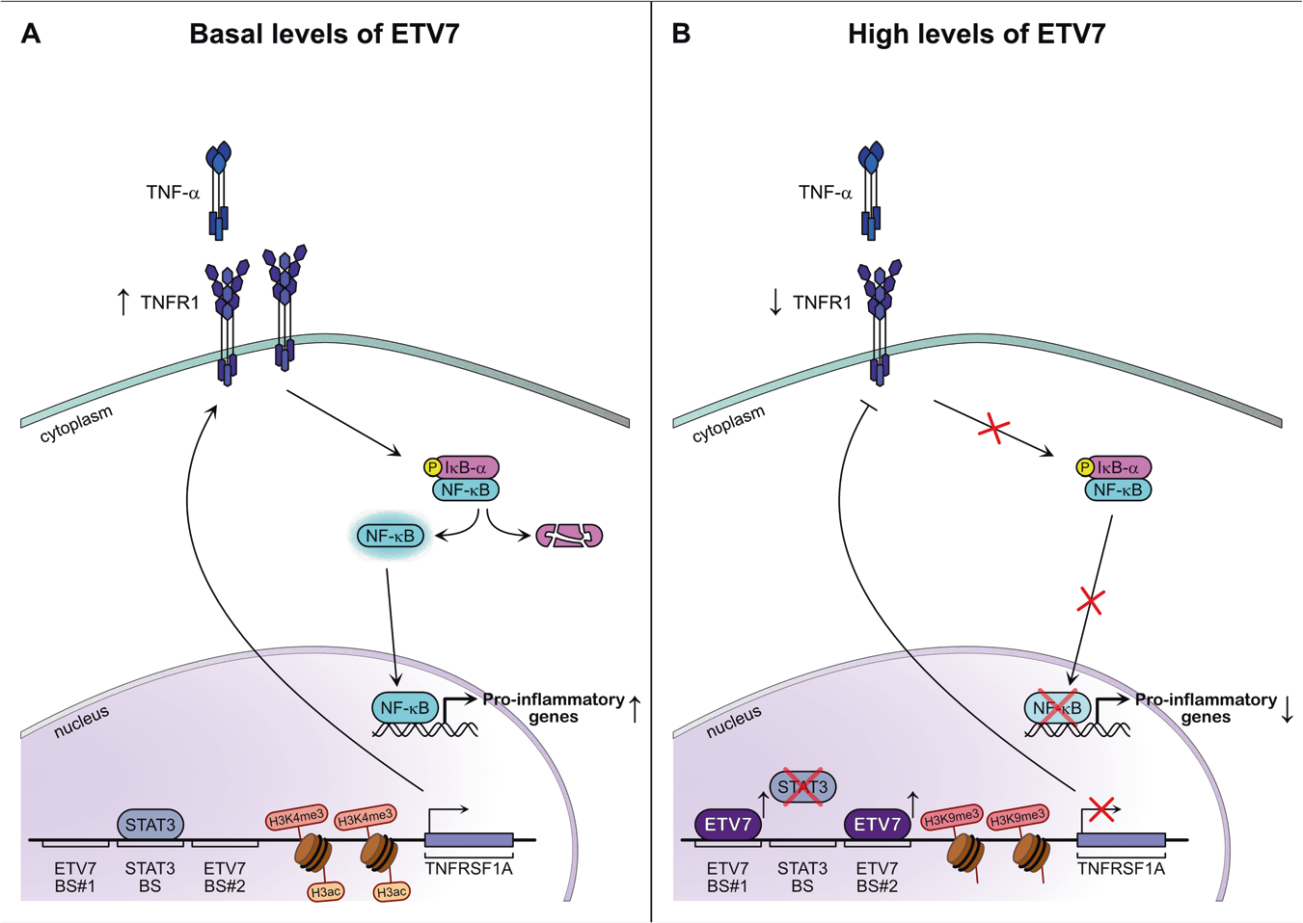
ETV7 can compete with STAT3 in the regulation of the *TNFRSF1A* gene influencing the NF-κB regulatory pathway. **A** The canonical STAT3/TNF-α/NF-κB regulatory pathway. STAT3 binds to its regulatory element in the first intron of the *TNFRSF1A* gene and induces its expression by recruiting chromatin remodelers that result in an “active” state. Consequently, the TNF-α receptor 1 is produced. TNF-α molecules bind the TNFR1 receptor and activate the NF-κB signaling pathway. **B** In the context where ETV7 expression is increased, ETV7 can displace STAT3 from its binding sites on the Intron 1 of TNFRSF1A and directly represses its expression by altering the deposition of histone marks. This ETV7-mediated repression leads to the reduced activation of NF-κB signaling and, hence, reduces the expression of pro-inflammatory genes.

However, to better characterize the biological impact of NF-κB target genes repression, the reduced expression of pro-inflammatory cytokines or chemokines should be investigated by performing secretome analysis in cells over-expressing ETV7 or the empty vector as a control. Nonetheless, with ELISA assay on supernatants of cells over-expressing ETV7, we have demonstrated that it can reduce the release of IL-6 and IL-8 pro-inflammatory cytokines even in response to TNF-α stimulation (Fig. 4D and Supplementary Fig. 3E). After introducing ectopic TNFR1 into our cellular systems, we confirmed that the reduced NF-κB activity was, at least partially, dependent on the ETV7-mediated repression of TNFRSF1A (Fig. 4E and Supplementary Fig. 3C, D). The partial response could be explained by the fact that ETV7 also represses other elements, such as Toll-like receptor 2 or IL-1 receptor 1, which are also involved in the activation of NF-κB [45, 47]. Collectively, our results suggest that the ETV7-mediated modulation of the TNFRSF1A expression regulates NF-κB activity in MCF7 and T47D cells. Based on our data, we propose that ETV7 represses TNFRSF1A through the displacement of STAT3 (working as a positive regulator) from TNFRSF1A Intron 1, the alteration of deposition of histone marks, which results in a significant reduction of NF-κB-dependent responses (Fig. 7).

The role of NF-κB in tumor cells and tumor microenvironment is ambivalent and is highly dependent on the tumor context. It is widely reported that NF-κB target genes control several pro-tumorigenic processes such as proliferation, cell survival, angiogenesis, and invasion. Furthermore, NF-κB sustains tumor-associated chronic inflammation by producing chemokines and cytokines. However, an increasing number of studies demonstrate the role of NF-κB as a tumor suppressor, particularly important in regulating the anti-tumor immune response. We hypothesize that the ETV7-dependent reduced activation of NF-κB could help cancer cells evade the host immune response, as proper stimulation is essential for both innate and adaptive immune responses [48]. For example, NF-κB controls the mRNA expression and protein stability of PD-L1 in tumor cells, thereby promoting the inhibition of cytotoxic CD8^+^ T cells [49, 50]. Besides, a study in a pancreatic ductal carcinoma mouse model shows that TNF and TNFR1 are required for optimal cytotoxic CD8^+^ T function and tumor rejection [51].

Furthermore, reduced activation of NF-κB leads to loss of MHC-I expression, which is one of the most important mechanisms of immune evasion in cancer [52, 53]. The loss of MHC-I, in turn, results in reduced sensitivity to immunotherapy. Finally, NF-κB has many autonomous functions in immune cells in the tumor microenvironment [54, 55]. Even though the effect of ETV7 on the cytotoxic CD8^+^ T cells and the response to the immunotherapy remains to be studied, previous data showing the repression of IFN response in cancer and viral infections, as well as the new data demonstrated in this study strongly suggest that ETV7 may play a role in the repression of the inflammatory and immune processes in breast cancer.

Taken collectively, our present study demonstrates that ETV7 represses TNFRSF1A expression by displacing STAT3 on its regulatory element. This down-regulation leads to the reduced activation of NF-κB signaling and thus suppresses the inflammatory and immune pathways in breast cancer cells.

## Materials and methods

### Cell lines and culture conditions

MCF7 were obtained from Interlab Cell Line Collection Bank (IRCCS Ospedale Policlinico San Martino, Genoa, Italy), and T47D cells were received from Dr. U. Pfeffer (IRCCS Ospedale Policlinico San Martino). MDA-MB-231 and SK-BR-3 cells were a gift from Prof. A. Provenzani (CIBIO Department, University of Trento, Italy). ZR-75-1 cells were obtained from Prof. A. Zippo (CIBIO Department).

As described previously [18], MCF7 and T47D cells were transduced with pAIP-ETV7 or pAIP-Empty lentiviral vectors to stably over-express ETV7. MCF7, SK-BR-3, and T47D were grown in DMEM medium (Gibco, ThermoFisher Scientific, Milan, Italy) supplemented with 10% of FBS (Gibco), 2mM L-Glutamine (Gibco) and a mixture of 1000U/ml Penicillin/100 µg/ml Streptomycin (Gibco); in the case of the stable over-expression with pAIP-ETV7/Empty plasmids, for the selection 0.75 µg/ml or 1.5 µg/ml of Puromycin (Gibco), respectively, was added to the medium. MDA-MB-231 cells were cultivated in DMEM medium (Gibco) supplemented with 10% of FBS (Gibco), 2mM L-Glutamine (Gibco), a mix of 1000U/ml Penicillin/100 µg/ml Streptomycin (Gibco), and 1% of Non-Essential Amino Acids (Gibco). ZR-75-1 cells were cultivated in RPMI medium (Gibco) supplemented with 10% FBS (Gibco), 2mM L-Glutamine (Gibco), a mixture of 100 U/ml Penicillin/100 µg/ml Streptomycin (Gibco) and 1% of Sodium pyruvate (Gibco). Cells were maintained in a humidified atmosphere at 37 °C with 5% of CO_2_. Cell lines were regularly monitored for mycoplasma contamination, were recently authenticated (i.e., by STR profiling), and passed for less than two months after thawing.

### Cytokine stimulation and treatment with chemotherapeutic agents

MCF7 and T47D cells were treated with 20 ng/ml IL-6 (PeproTech, London, UK) and 10 ng/ml or 20 ng/ml TNF-α (PeproTech), respectively. Cells were stimulated for 1 h for protein analysis and immunofluorescence, 4 h for Chromatin ImmunoPrecipitation, luciferase reporter assays, and mRNA analyses, and 24 h for the preparation of conditioned medium for ELISA. MCF7, T47D, MDA-MB-231, and SK-BR-3 cells were treated with 1.5 µM Doxorubicin (MedChemExpress -MCE-, D.B.A. Italia, Milan, Italy) and 375 μM of 5-fluorouracil (5-FU) (Sigma Aldrich, Milan, Italy) for 24 h for mRNA analyses and Chromatin Immunoprecipitation.

### RNA extraction and RT-qPCR

Total RNA was isolated using the RNeasy Mini Kit (Qiagen, Milan, Italy) and converted into cDNA with the PrimeScriptTM RT reagent kit (Takara, Diatech Lab Line, Ancona, Italy). RT-qPCR was performed with 25 ng of the template cDNA using the qPCRBIO SyGreen 2X master mix (PCR Biosystems, Resnova, Rome, Italy) and CFX384 (Biorad, Milan, Italy) was used as a detection system. YWHAZ and ACTB were used as housekeeping genes. Relative fold change was calculated using the ΔΔCt method [56] as described previously [57]. For primer design, the Primer-BLAST online tool [58] was used, and all primers (obtained both from Eurofins Genomics, Ebersberg, Germany, and Metabion International AG, Planegg/Steinkirchen, Germany) were tested for specificity and efficiency. Primer sequences are listed in Supplementary Table 1.

### Western blot

Total protein cell extracts were obtained by lysing the cells with RIPA buffer supplemented with 1x protease inhibitors (PI) (Roche, Milan, Italy). Proteins were quantified using the BCA method (Pierce, ThermoFisher Scientific), and then 30–50 µg of proteins were loaded on 8–12% polyacrylamide gels for SDS-PAGE. After the separation, the proteins were transferred on a nitrocellulose membrane (Amersham, Merck,) which was probed over-night at 4 °C with specific antibodies diluted in 1–3% skimmed milk-PBS-0.1% Tween solution: TNFR1 (H-5, Santa Cruz Biotechnologies, DBA, Milan, Italy), STAT3 (124H6, Cell Signaling Technologies, Euroclone, Milan, Italy), pSTAT3 (Y705, Cell Signaling Technologies), HSP70 (C92F3A-5, Santa Cruz Biotechnologies), TEL2 (E-1, Santa Cruz Biotechnologies), GAPDH (6C5, Santa Cruz Biotechnologies), H3 (Abcam), pIκBα (Ser32/36, Santa Cruz Biotechnologies). Detection was performed with ECL Select Reagent (GE Healthcare, Cytiva) using UVITec Alliance LD2 (UVITec Cambridge, UK) imaging system.

### Cytoplasmic-nuclear fractionation

Cytosolic and chromatin-enriched protein fractions were extracted as recently described [59]. Briefly, cellular pellets were resuspended in NSB buffer (10 mM HEPES, 10 mM KCl, 1.5 mM MgCl_2_, 0.34 M Sucrose, 10% Glycerol, 1 mM DTT, 0.1% TritonX100 added with 1x PI and 1x Phosphatase Inhibitors - Roche) and left on ice for 8 min. Then the samples were centrifuged at 1300 rpm at 4 °C for 10 min. The supernatant containing the cytoplasmic protein fraction was collected. The remaining nuclei were resuspended in the NSB buffer supplemented with 1 mM CaCl_2_ and 2000 gel units/ml of MNase (New England Biolabs) and incubated at 37 °C for 10 min. The MNase reaction is stopped by adding 2 mM EGTA (Sigma‐Aldrich/Merck). Afterward, the samples were centrifuged at 13,000 rpm at 4 °C for 10 min, and the fraction containing the nuclear-soluble proteins was collected. The remaining pellet was resuspended in the NSB buffer supplemented with 600 mM NaCl and incubated at 4 °C overnight. Then, the samples were centrifuged at 13,000 rpm at 4 °C for 10 min, and chromatin-enriched protein fraction was collected. The protein samples were then loaded on the SDS-PAGE gel, and the Western blot procedure was executed as described above.

### Plasmids and cloning

The expression plasmids pCMV6-Entry-Empty and pCMV6-Entry-ETV7 C-terminally tagged with DDK-Myc were purchased from Origene (Tema Ricerca, Bologna, Italy). pGL3-NF-κb reporter, containing the *Photinus pyralis* (Firefly) luciferase gene under the control of an NF-κB responsive element, was a gift from Dr. Alessio Nencioni (University of Genoa, Italy). 4xM67 pTATA TK-Luc, containing four copies of the sequence GGTTCCCGTAAATGCATCA (underlined is the STAT-binding site), was obtained from Prof. David Frank (Dana-Farber Cancer Institute, Boston, CA, USA). pRL-SV40 (Promega) plasmid constitutively expressing the *Renilla reniformis* luciferase cDNA was used as transfection efficiency control for the gene reporter assays.

The pcDNA3-TNFR1 expression vector was generated by cloning with the primers indicated below to PCR amplify (using Q5 High-Fidelity PCR kit, New England Biolabs, Euroclone, Milan, Italy) the TNFR1 reference sequence from pBMNZ-neo-Flag-TNFR1 L380A (gift from Martin Kluger, Addgene plasmid # 43949; http://n2t.net/addgene:43949) [60] and inserting it into pcDNA3.1 plasmid (the tails containing the target sequences of restriction endonucleases are indicated in lowercases):

Fw: gcggtaccATGAGGGCCTGGATCTTCTTTC

Rv: tagcggccgcTCATCTGAGAAGACTGGGCGCG

The purified PCR product was inserted into the pcDNA3.1 backbone using KpnI and NotI restriction endonucleases and T4 DNA Ligase (New England Biolabs). Correct cloning was verified by diagnostic restriction and direct sequencing (Microsynth, Balgach, Switzerland).

### Transient transfections

24 h prior transfection, 0.2 ×10^6^ SK-BR3, MDA-MB-231, and BT549 cells were seeded in 6-well plates. Cells were transfected using Lipofectamine LTX and Plus Reagent (Life Technologies) along with 1 µg of pCMV-Entry-Empty or pCMV-Entry-ETV7 plasmid (Origene and our previous study [19]). After 48 h, the cells were collected and processed accordingly.

### Gene reporter assays

First, 90,000 cells per well were seeded in 24-well plates, and after 24 h, the cells were transfected with Lipofectamine LTX and Plus Reagent (ThermoFisher Scientific) along with different combinations of the plasmids according to the experiment: 50 ng of normalizing vector pRL-SV40, 200 ng of expression vectors (pcDNA3.1-Empty/pcDNA3.1-TNFR1), and reporter vectors (350 ng 4xM67 pTATA TK-Luc and 300 ng pGL3-NF-κB). Twenty-four hours post-transfection, if necessary, the cells were stimulated with appropriate concentrations of different cytokines. Then, the cells were washed once with 1X PBS and lysed in 1X PLB (Passive Lysis Buffer) buffer (Promega). Afterward, the luciferase activity was measured using the Dual-Luciferase Reporter Assay System (Promega) following the manufacturer’s procedure and using the Varioskan LUX multimode microplate reader (ThermoFisher Scientific). *Renilla* luciferase activity was used as an indicator of transfection efficiency and to obtain the Relative Light Unit (RLU) values as previously described [61].

### RNA interference (siRNA)

Small interfering RNAs (siRNAs) and the transfection reagent INTERFERin® (Polyplus-Transfection, Euroclone) were used to reduce the expression of target RNAs. Scrambled siRNA was used as a control. Scrambled siRNA and ETV7 targeting siRNA (#1 and #2) were purchased from Integrated DNA Technologies (IDT, Tema Ricerca). 24 h before transfection, cells were seeded in 6-well plates to reach 60–70% confluence. Then, cells were transfected with 20 nM siRNA and 8 μl INTERFERin reagent per well. The transfection mix was diluted in 200 μl OptiMEM medium (Gibco, Life Technologies), vortexed for 10 s, incubated at room temperature for 15 min, and drop by drop added to cells. Analyses on the silenced cells were performed 72 h post-transfection.

### Chromatin immunoprecipitation (ChIP)

ChIP experiments were performed as previously described [19]. Briefly, 3 × 10^6^ MCF7 Empty/ETV7 or T47D Empty/ETV7 cells were seeded in 15 cm dishes. The day after, if necessary, the cells were treated with 20 ng/ml IL-6 for 4 h or with 1.5 µM Doxorubicin for 24 h and afterward cross-linked for 8 min using 1% Formaldehyde. At the end of the incubation, 125 mM Glycine was added and left for 5 min. Then, the cells were washed with ice-cold 1X PBS, scraped, and collected in 1X PBS supplemented with protease inhibitors (PI). Then, the pellet was lysed with lysis buffer (1% SDS) supplemented with 100 µg/ml salmon sperm single-strand DNA (ssDNA) and PI. After the lysis, the samples were centrifuged, and the supernatant was discarded. Then, the pellets were resuspended in the sonication buffer (0.25% SDS, 200 mM NaCl) supplemented with 100 µg/ml ssDNA and PI and sonicated using Bioruptor Pico sonicator (Diagenode, Denville, NJ, USA). In order to reach DNA fragments in the range of 200–700 bp, for the MCF7 cells, we used 45 cycles (30 s On/ 30 s Off), and for T47D cells, 15 cycles (30 s On/30 s Off). Then, the samples were diluted and incubated with 2 µg the appropriate antibody targeting ETV7 (Santa Cruz, TEL2, E-1), pSTAT3 (Cell Signaling Technologies, 124H6), H3K9me3 (Cell Signaling Technologies, 13969P), H3K4me3 (Abcam, ab8580), H3ac (Abcam, ab47919) or IgG (Santa Cruz Biotechnologies, mouse or rabbit according to the antibody used) and Dynabeads with protein G or A (Life Technologies) overnight at 4 °C in a rotator. The input sample (10% of the sample volume) was incubated overnight at 4 °C without dilution or addition of any antibodies or beads. The day after, the samples were washed through multiple washing steps and eluted at 65 °C overnight by adding the elution buffer, and 1X TE supplemented with 0.65% of SDS. Then the samples were processed with 50 µg of Proteinase K (ThermoFisher Scientific) for 2 h at 56 °C and 50 µg of RNase A (VWR International, Radnor, PA, USA) for 30 min at 37 °C. Afterward, DNA was purified using a QIAquick PCR purification kit (Qiagen, Germany). qPCR was performed using GoTaq® qPCR Master Mix (Promega) and BioRad CFX384 qPCR system. Primer sequences are listed in Supplementary Table 1.

### Co-Immunoprecipitation

MCF7-Empty/ETV7 or T47D-Empty/ETV7 cells were seeded in P100 dishes. After 24 h, cells were treated with 20 ng/ml IL-6 and 4 h post-treatment lysed using CHAPS buffer and incubated overnight with 2 µg of an anti-ETV7 antibody (TEL2, Santa Cruz Biotechnologies) or normal mouse IgG (Santa Cruz Biotechnologies) previously bound with Dynabeads protein G magnetic beads (Life Technologies). Then, the beads were washed, and the immunoprecipitated lysates were eluted and loaded on a polyacrylamide gel for SDS-PAGE. The following steps were performed equally to the previously described Western blot procedure.

### Immunofluorescence

Cells after the stimulation with 10 ng/ml TNF-α (60 min) were fixed with 4% PFA (Sigma-Aldrich) and incubated for 10 min at room temperature. Afterward, wells were washed once with 100 µl of 1X PBS. Then, the cells were blocked and permeabilized using 3%BSA-0.3%Triton-X-100/PBS solution for 30 min at room temperature. Primary anti-p65 (NF-κB p65, clone D14E12, XP, Cell Signaling Technologies) antibody was diluted 1:400 in 1% BSA solution and added to the wells. Afterwards, the plate was incubated for 60 min at room temperature, and the wells were washed once with 3% BSA solution. Then, the anti-Rabbit IgG (H + L) Cross-Adsorbed Secondary Antibody-Alexa Fluor® 488 (Life Technologies) was diluted 1:500 in 1% BSA solution, added to the wells, and incubated for 60 min at room temperature protected from the light. Afterward, the wells were washed once with 3% BSA solution. The nuclei were stained using Hoechst 33342 (1:5000) and incubated for 30 min at room temperature. The images were acquired using ImageXpress MD Micro Confocal High-Content Imaging System (20X).

### Conditioned medium and ELISA

Cells were seeded in 10 cm dish, and after reaching the 70-80% confluency cells were washed once with 1X PBS, and new medium containing reduced FBS (2.5%) was added. Cells were left growing for 48 h, conditioned medium was collected, filtered using 0.45 µm syringe filter and stored at −20 °C for a maximum of 2 weeks before the ELISA assay. When the cells needed to be stimulated, 10 ng/ml of TNF-α was added 24 h before the collection of conditioned medium.

ELISA assays were performed using AuthentiKine^TM^ kits (Proteintech®, D.B.A. Italia) for TNF-α, IL-8, and IL-6. Shortly, 100 µl of cell supernatant or standard solutions were added to wells and the plate was incubated for 2 h at 37 °C. After the incubation, wells were thoroughly washed 4 times with wash buffer. Afterwards, 100 µl of the diluent antibody solution (1:100 for IL-6 and IL-8, and 1:75 for TNF-α) were added to each well and the plate was incubated for 1 h, at 37 °C. The cycle of washes was performed again. Then, 100 µl of diluent HRP solution (1:100) were added to each well and the plate was incubated for 40 min at 37 °C. The cycle of washes was performed another time. Afterwards, 100 µl of TMB solution was added to each well and the plate was incubated for 20 min at 37 °C protected from the light. The reaction was stopped by adding 100 µl of stop solution. The absorbance was measured immediately at 450 nm and 630 nm, using the Varioskan LUX multimode microplate reader (ThermoFisher Scientific).

### Gene expression profiling

The list of differentially regulated genes and enrichment scores for gene function and biological processes were obtained as we described in our recent study [18]. Data were deposited with the accession number GSE152580 on the Gene Expression Omnibus database (GEO, https://www.ncbi.nlm.nih.gov/geo/). GSEA and GO results are available from our previous work [18].

### Statistics

If not indicated otherwise, statistical analyses were performed using GraphPad Prism version 9 software. For determining the statistical significance among two classes of samples, the unpaired Student’s *t* test was used. Graphic illustrations were generated using the Affinity designer tool (Serif, West Bridgford, UK).

### Analysis of breast cancer patients’ data

#### From TCGA

gene expression data (raw counts) and clinical information of 1102 primary tumors and 112 paired normal tissues from the breast cancer TCGA dataset (TCGA BRCA) were downloaded from the Genomic Data Commons Portal using functions of the TCGAbiolinks R package (version 2.22.4). Raw counts were normalized and gene expression levels quantified as counts per million (cpm) using functions of the edgeR R package (version 3.36.0). The set of genes regulated by NF-kB in response to TNF (TNFA_SIGNALING_VIA_NFKB) and defining inflammatory response (INFLAMMATORY_RESPONSE) have been downloaded from the Hallmark collection of the Molecular Signatures Database (MSigDB v2022.1.Hs; http://www.gsea-msigdb.org/gsea/msigdb/human/genesets.jsp?collection=H). Expression levels of genes and gene sets in paired primary tumors and normal tissues and in the molecular subtypes of primary tumors have been compared using the parametric test of the *ggwithinstats* and *ggbetweenstats* functions of the *ggstatsplot* R package (version 0.10.0), respectively. To identify two groups of tumors with either high or low level of genes regulated by NF-κB in response to TNF and of genes defining inflammatory response, we used the classifier described by Adorno and colleagues [62], that is a classification rule based on gene expression signature scores. Briefly, the signature scores have been obtained summarizing the standardized expression levels of TNFA_SIGNALING_VIA_NFKB and INFLAMMATORY_RESPONSE genes into a combined score with zero mean. Tumors were classified as signature ‘Low’ if the combined score was smaller than the median signature score and as signature ‘High’ vice versa. This classification was applied to the expression values of the TCGA BRCA primary tumors with survival information (n = 1100). To evaluate the prognostic value of the signatures, we estimated, using the Kaplan–Meier method, the probabilities of disease-specific survival. To confirm these findings, the Kaplan–Meier curves were compared using the log-rank (Mantel–Cox) test. P-value was calculated according to the standard normal asymptotic distribution. Survival analysis was performed using functions of *survival* (version 3.4-0) and *survminer* (version 0.4.9) packages.

#### From a private cohort

ETV7 and TNFRSF1A mRNA expression was analyzed in available microarray data from 197 breast cancer samples from the University Medical Center Hamburg-Eppendorf (Germany) [63]. Patients included in this cohort were treated between 1991 and 2002 and selected on the basis of tissue availability. Informed consent for the scientific use of tissue materials, which was approved by the local ethics committees (for Hamburg: Ethik-Kommission der Ärztekammer Hamburg, #OB/V/03), was obtained from all patients. The study was performed in accordance with the principles of the declaration of Helsinki and REMARK criteria [64]. No radiotherapy, neoadjuvant chemotherapy or endocrine therapy had been administered before surgery. Patient characteristics are described in Supplementary Table 2.

The Affymetrix chip used in this analysis (Affymetrix HG-U133A array, Santa Clara, CA, USA) harbors one probeset for ETV7 (221680_s_at) and one probeset for TNFRSF1A (207643_s_at). The cohort was divided into quartiles according to their expression values, and Kaplan–Meier analyses with log-rank tests were performed and visualized with the most suitable cut-off (Q1 = lowest 25% vs. Q2-Q4 = higher 75%) using the SPSS 27.0 software. Correlations between ETV7 and TNFRSF1A mRNA levels were calculated by Chi-square tests using the SPSS 27.0 software and visualized using GraphPad Prism™ 9 software (San Diego, CA, USA). All tests were performed at a significance level of p = 0.05.

#### From a second private cohort

ETV7 and TNFRSF1A mRNA expression was analyzed from online available RNA-seq data with accession number GSE58135 from Gene Expression Omnibus repository [65]. This patient cohort includes 42 Estrogen Receptor-positive breast cancers (ER+), 42 Triple-Negative breast cancers (TNBC), and, respectively, 30 and 21 matched normal adjacent tissues. Significance was calculated with Student’s *t* test by comparing two groups of samples at a time.

Additional data from cancer patients were obtained from available online tools; specifically, to determine the correlation between TNFRSF1A expression levels and prognosis in breast cancer patients we used Kaplan–Meier plotter (http://kmplot.com/analysis/, [66]).

### Immunohistochemistry (IHC)

On a few clinical samples from Santa Chiara Hospital, APSS, Trento, IHC was performed in a Bond Max Automated Immunohistochemistry Vision Biosystem (Leica Microsystems, Wetzlar, Germany) using the Bond Polymer Refine Detection kit (DS9800; Leica Biosystems, Wetzlar, Germany) as previously described [67]. Briefly, 3-µm-thick sections were prepared from formalin-fixed paraffin-embedded tissue blocks, deparaffinized, pre-treated with epitope retrieval solution 2 (pH9; Leica Biosystems) at 100 °C for 20 min, and then incubated for 30 min with primary antibodies (ETV7/TEL2, E-1, 1:50; TNFR1, H-5, 1:50, Santa Cruz Biotechnology) diluted in Bond Primary Antibody Diluent (AR9352; Leica Biosystems). All the slides were reviewed by a pathologist. Informed consent for use of tissue materials for research purposes was approved by the local ethics committee (APSS, Trento, Italy) and was obtained from all subjects.

## Supplementary information

Reproducibility checklist

Supplementary Figure Legends

Supplementary Figure 1

Supplementary Figure 2

Supplementary Figure 3

Supplementary Figure 4

Supplementary Table 1

Supplementary Table 2

Original WB

Authorship form

## Data Availability

The data and the resources of this study are available from the corresponding author (yari.ciribilli@unitn.it) upon reasonable request. Additional data are available as supplementary material.

## References

[1] Siegel RL, Miller KD, Jemal A (2020). Cancer statistics, 2020. CA: Cancer J Clin..

[2] Bray F, Ferlay J, Soerjomataram I, Siegel RL, Torre LA, Jemal A (2018). Global cancer statistics 2018: GLOBOCAN estimates of incidence and mortality worldwide for 36 cancers in 185 countries. CA: Cancer J Clin.

[3] Gonzalez-Angulo AM, Morales-Vasquez F, Hortobagyi GN (2007). Overview of resistance to systemic therapy in patients with breast cancer. Adv. Exp. Med. Biol.

[4] Dillekas H, Rogers MS, Straume O (2019). Are 90% of deaths from cancer caused by metastases?. Cancer Med.

[5] Riggio AI, Varley KE, Welm AL (2021). The lingering mysteries of metastatic recurrence in breast cancer. Br J Cancer.

[6] Greten FR, Grivennikov SI (2019). Inflammation and cancer: triggers, mechanisms, and consequences. Immunity..

[7] Kalliolias GD, Ivashkiv LB (2016). TNF biology, pathogenic mechanisms and emerging therapeutic strategies. Nat Rev Rheumatol.

[8] Ting AT, Bertrand MJM (2016). More to life than NF-kappaB in TNFR1 signaling. Trends Immunol.

[9] Mercogliano MF, Bruni S, Elizalde PV, Schillaci R (2020). Tumor necrosis factor alpha blockade: an opportunity to tackle breast cancer. Front. Oncol.

[10] Balkwill F (2009). Tumour necrosis factor and cancer. Nat Rev Cancer.

[11] O’Reilly LA, Putoczki TL, Mielke LA, Low JT, Lin A, Preaudet A (2018). Loss of NF-kappaB1 causes gastric cancer with aberrant inflammation and expression of immune checkpoint regulators in a STAT-1-dependent manner. Immunity..

[12] Cornel AM, Mimpen IL, Nierkens S (2020). MHC class I downregulation in cancer: underlying mechanisms and potential targets for cancer immunotherapy. Cancers (Basel).

[13] Carella C, Potter M, Bonten J, Rehg JE, Neale G, Grosveld GC (2006). The ETS factor TEL2 is a hematopoietic oncoprotein. Blood..

[14] Li H, Zhang Y, Zheng S (2021). Comprehensive analysis Identified ETV7 as a potential prognostic biomarker in bladder cancer. BioMed Res Int.

[15] Matos JM, Witzmann FA, Cummings OW, Schmidt CM (2009). A pilot study of proteomic profiles of human hepatocellular carcinoma in the United States. J Surg Res.

[16] Piggin CL, Roden DL, Gallego-Ortega D, Lee HJ, Oakes SR, Ormandy CJ (2016). ELF5 isoform expression is tissue-specific and significantly altered in cancer. Breast Cancer Res: BCR.

[17] Harwood FC, Klein Geltink RI, O’Hara BP, Cardone M, Janke L, Finkelstein D (2018). ETV7 is an essential component of a rapamycin-insensitive mTOR complex in cancer. Sci Adv..

[18] Pezze L, Meskyte EM, Forcato M, Pontalti S, Badowska KA, Rizzotto D (2021). ETV7 regulates breast cancer stem-like cell features by repressing IFN-response genes. Cell Death Dis.

[19] Alessandrini F, Pezze L, Menendez D, Resnick MA, Ciribilli Y (2018). ETV7-mediated DNAJC15 repression leads to doxorubicin resistance in breast cancer cells. Neoplasia..

[20] Bisio A, Zamborszky J, Zaccara S, Lion M, Tebaldi T, Sharma V (2014). Cooperative interactions between p53 and NFkappaB enhance cell plasticity. Oncotarget..

[21] Minutti CM, Garcia-Fojeda B, Saenz A, de Las Casas-Engel M, Guillamat-Prats R, de Lorenzo A (2016). Surfactant protein A prevents IFN-gamma/IFN-gamma receptor interaction and attenuates classical activation of human alveolar macrophages. J Immunol.

[22] Qiao Y, Kang K, Giannopoulou E, Fang C, Ivashkiv LB (2016). IFN-gamma induces histone 3 lysine 27 trimethylation in a small subset of promoters to stably silence gene expression in human macrophages. Cell Rep.

[23] Matz M, Heinrich F, Zhang Q, Lorkowski C, Seelow E, Wu K (2018). The regulation of interferon type I pathway-related genes RSAD2 and ETV7 specifically indicates antibody-mediated rejection after kidney transplantation. Clin Transplant.

[24] Pervolaraki K, Rastgou Talemi S, Albrecht D, Bormann F, Bamford C, Mendoza JL (2018). Differential induction of interferon stimulated genes between type I and type III interferons is independent of interferon receptor abundance. PLoS Pathogens.

[25] Froggatt HM, Harding AT, Chaparian RR, Heaton NS (2021). ETV7 limits antiviral gene expression and control of influenza viruses. Sci Signal.

[26] Chaudhuri S, Thomas S, Munster P (2021). Immunotherapy in breast cancer: a clinician’s perspective. J Natl Cancer Center..

[27] Li Y, Miao W, He D, Wang S, Lou J, Jiang Y (2021). Recent progress on immunotherapy for breast cancer: tumor microenvironment, nanotechnology and more. Front Bioeng Biotechnol.

[28] Potter MD, Buijs A, Kreider B, van Rompaey L, Grosveld GC (2000). Identification and characterization of a new human ETS-family transcription factor, TEL2, that is expressed in hematopoietic tissues and can associate with TEL1/ETV6. Blood..

[29] Gu X, Shin BH, Akbarali Y, Weiss A, Boltax J, Oettgen P (2001). Tel-2 is a novel transcriptional repressor related to the Ets factor Tel/ETV-6. J Biol Chem.

[30] Wei GH, Badis G, Berger MF, Kivioja T, Palin K, Enge M (2010). Genome-wide analysis of ETS-family DNA-binding in vitro and in vivo. EMBO J.

[31] Egusquiaguirre SP, Yeh JE, Walker SR, Liu S, Frank DA (2018). The STAT3 target gene TNFRSF1A modulates the NF-kappaB pathway in breast cancer cells. Neoplasia..

[32] Raskatov JA, Meier JL, Puckett JW, Yang F, Ramakrishnan P, Dervan PB (2012). Modulation of NF-kappaB-dependent gene transcription using programmable DNA minor groove binders. Proc Natl Acad Sci USA.

[33] Kanarek N, London N, Schueler-Furman O, Ben-Neriah Y (2010). Ubiquitination and degradation of the inhibitors of NF-kappaB. Cold Spring Harb Perspect Biol.

[34] Kanarek N, Ben-Neriah Y (2012). Regulation of NF-kappaB by ubiquitination and degradation of the IkappaBs. Immunol Rev.

[35] Vallania F, Schiavone D, Dewilde S, Pupo E, Garbay S, Calogero R (2009). Genome-wide discovery of functional transcription factor binding sites by comparative genomics: the case of Stat3. Proc Natl Acad Sci USA.

[36] Tripathi SK, Chen Z, Larjo A, Kanduri K, Nousiainen K, Aijo T (2017). Genome-wide analysis of STAT3-mediated transcription during early human Th17 cell differentiation. Cell Rep.

[37] Qu H, Zhao H, Zhang X, Liu Y, Li F, Sun L (2020). Integrated analysis of the ETS family in melanoma reveals a regulatory role of ETV7 in the immune microenvironment. Front. Immunol.

[38] Mantovani A, Barajon I, Garlanda C (2018). IL-1 and IL-1 regulatory pathways in cancer progression and therapy. Immunol Rev.

[39] Hanna BS, Llao-Cid L, Iskar M, Roessner PM, Klett LC, Wong JKL (2021). Interleukin-10 receptor signaling promotes the maintenance of a PD-1(int) TCF-1(+) CD8(+) T cell population that sustains anti-tumor immunity. Immunity..

[40] Mokhtari Y, Pourbagheri-Sigaroodi A, Zafari P, Bagheri N, Ghaffari SH, Bashash D (2021). Toll-like receptors (TLRs): An old family of immune receptors with a new face in cancer pathogenesis. J Cell Mol Med.

[41] Boccuni P, MacGrogan D, Scandura JM, Nimer SD (2003). The human L(3)MBT polycomb group protein is a transcriptional repressor and interacts physically and functionally with TEL (ETV6). J Biol Chem.

[42] Jackson-Bernitsas DG, Ichikawa H, Takada Y, Myers JN, Lin XL, Darnay BG (2007). Evidence that TNF-TNFR1-TRADD-TRAF2-RIP-TAK1-IKK pathway mediates constitutive NF-kappaB activation and proliferation in human head and neck squamous cell carcinoma. Oncogene..

[43] Wajant H, Scheurich P (2011). TNFR1-induced activation of the classical NF-kappaB pathway. FEBS J.

[44] Soleimani A, Rahmani F, Ferns GA, Ryzhikov M, Avan A, Hassanian SM (2020). Role of the NF-kappaB signaling pathway in the pathogenesis of colorectal cancer. Gene..

[45] Bonizzi G, Karin M (2004). The two NF-kappaB activation pathways and their role in innate and adaptive immunity. Trends Immunol.

[46] Taniguchi K, Karin MNF-kappaB (2018). inflammation, immunity and cancer: coming of age. Nat Rev Immunol.

[47] Yu H, Lin L, Zhang Z, Zhang H, Hu H (2020). Targeting NF-kappaB pathway for the therapy of diseases: mechanism and clinical study. Signal Transduct Target Ther.

[48] Pfeffer LM (2011). The role of nuclear factor kappaB in the interferon response. J Interferon Cytokine Res.

[49] Manguso RT, Pope HW, Zimmer MD, Brown FD, Yates KB, Miller BC (2017). In vivo CRISPR screening identifies Ptpn2 as a cancer immunotherapy target. Nature..

[50] Pan D, Kobayashi A, Jiang P, Ferrari de Andrade L, Tay RE, Luoma AM (2018). A major chromatin regulator determines resistance of tumor cells to T cell-mediated killing. Science..

[51] Chopra M, Lang I, Salzmann S, Pachel C, Kraus S, Bauerlein CA (2013). Tumor necrosis factor induces tumor promoting and anti-tumoral effects on pancreatic cancer via TNFR1. PLoS ONE.

[52] Jongsma MLM, Guarda G, Spaapen RM (2019). The regulatory network behind MHC class I expression. Mol Immunol.

[53] Dhatchinamoorthy K, Colbert JD, Rock KL (2021). Cancer immune evasion through loss of MHC class I antigen presentation. Front Immunol.

[54] Dorrington MG, Fraser IDC (2019). NF-kappaB signaling in macrophages: dynamics, crosstalk, and signal integration. Front Immunol.

[55] Lalle G, Twardowski J, Grinberg-Bleyer Y. NF-kappaB in cancer immunity: friend or foe? Cells. 2021;10:355. 10.3390/cells10020355 PMC7914614 33572260

[56] Pfaffl MW (2001). A new mathematical model for relative quantification in real-time RT-PCR. Nucleic Acids Res.

[57] Monti P, Ciribilli Y, Bisio A, Foggetti G, Raimondi I, Campomenosi P (2014). N-P63alpha and TA-P63alpha exhibit intrinsic differences in transactivation specificities that depend on distinct features of DNA target sites. Oncotarget..

[58] Ye J, Coulouris G, Zaretskaya I, Cutcutache I, Rozen S, Madden TL (2012). Primer-BLAST: a tool to design target-specific primers for polymerase chain reaction. BMC Bioinformatics.

[59] Tadijan A, Precazzini F, Hanzic N, Radic M, Gavioli N, Vlasic I (2021). Altered expression of shorter p53 family isoforms can impact melanoma aggressiveness. Cancers (Basel).

[60] D’Alessio A, Kluger MS, Li JH, Al-Lamki R, Bradley JR, Pober JS (2010). Targeting of tumor necrosis factor receptor 1 to low density plasma membrane domains in human endothelial cells. J Biol Chem.

[61] Bisio A, Latorre E, Andreotti V, Bressac-de Paillerets B, Harland M, Scarra GB (2015). The 5’-untranslated region of p16INK4a melanoma tumor suppressor acts as a cellular IRES, controlling mRNA translation under hypoxia through YBX1 binding. Oncotarget..

[62] Adorno M, Cordenonsi M, Montagner M, Dupont S, Wong C, Hann B (2009). A Mutant-p53/Smad complex opposes p63 to empower TGFbeta-induced metastasis. Cell..

[63] Milde-Langosch K, Karn T, Schmidt M, zu Eulenburg C, Oliveira-Ferrer L, Wirtz RM (2014). Prognostic relevance of glycosylation-associated genes in breast cancer. Breast Cancer Res Treat.

[64] McShane LM, Altman DG, Sauerbrei W, Taube SE, Gion M, Clark GM (2006). REporting recommendations for tumor MARKer prognostic studies (REMARK). Breast Cancer Res Treat.

[65] Varley KE, Gertz J, Roberts BS, Davis NS, Bowling KM, Kirby MK (2014). Recurrent read-through fusion transcripts in breast cancer. Breast Cancer Res Treat.

[66] Gyorffy B (2021). Survival analysis across the entire transcriptome identifies biomarkers with the highest prognostic power in breast cancer. Comput Struct Biotechnol J..

[67] Colaluca IN, Basile A, Freiburger L, D’Uva V, Disalvatore D, Vecchi M (2018). A Numb-Mdm2 fuzzy complex reveals an isoform-specific involvement of Numb in breast cancer. J Cell Biol.

