# Supplementary material for: ETV7 reduces inflammatory responses in breast cancer cells by repressing the TNFR1/NF-κB axis
Source: Cell Death Dis. 2023 Apr 12;14(4):263. doi: 10.1038/s41419-023-05718-y (PMC10089821; doi:10.1038/s41419-023-05718-y)
Supplement: Supplementary file 2 — Supplementary Figure Legends [file 41419_2023_5718_MOESM2_ESM.docx]

**Supplementary Figure 1.** A) A Gene Set Enrichment Analysis of T47D cells over-expressing ETV7 or its Empty counterpart. Enrichment plot for the inflammatory response in T47D cells (on the left) and TNFA signaling via NF-κB in T47D cells (on the right) gene sets of the Hallmark Collection. The Normalized Enrichment Score (NES) shows the degree of the enrichment of the gene set; the negative sign indicates that the gene set is down-regulated in cells over-expressing ETV7. FDR = False Discovery Rate. B) Western blot analysis of TNFR-1 protein in MDA-MB-231 and SK-BR-3 cells transiently over-expressing ETV7 or its empty counterpart. On the right of each blot is indicated the approximate observed molecular weight. HSP70 was used as a loading control. C-D) RT-qPCR analysis of the expression of the *TNFRSF1A* gene after the treatment with 1.5 µM Doxorubicin and 375 μM 5-fluorouracil for 24 h in MCF7 and T47D (C) and MDA-MB-231 and SK-BR-3 parental cells (D). Bars represent the averages and standard deviations of at least 3 biological replicates. E-F) RT-qPCR analysis of the expression of ETV7 after the treatment with 1.5 µM Doxorubicin (F) and 375 μM of 5-fluorouracil (E) for 24 h in MCF7, T47D, MDA-MB-231, and SK-BR-3 parental cells. Bars represent the averages and standard deviations of at least 3 biological replicates. G-J) Western blot analyses of ETV7 expression in MCF7, T47D, MDA-MB-231, and SK-BR-3 cells transfected with siRNA #1 and siRNA # 2 targeting ETV7 and the relative scramble control for 72 hours. HSP70 was used as a loading control. On the right of each blot is indicated the approximate observed molecular weight. K) Left: a dot plot demonstrating the differential expression analysis for the ETV7 gene in a BRCA-matched patients’ dataset from TCGA (The Cancer Genome Atlas) database. Tumor (light red), Normal (light blue). Right: a dot plot of ETV7 expression in ER-positive and triple-negative breast cancer patient cohort when comparing tumor tissue (light red) with matched adjacent tissue (light blue). ER+ - Estrogen Receptor-positive; TNBC – triple-negative breast cancer; Adj – adjacent tissue. Indicated are the means and the SEMs. L) A dot plot demonstrating the differential expression analysis for IL1R1 and TLR2 genes in a BRCA-matched patients’ dataset from TCGA (The Cancer Genome Atlas) database. Tumor (light red), Normal (light blue). M) Kaplan-Meier curves for RFS from a private breast cancer cohort according to the relative expression of TNFRSF1A. The number of patients and the statistical analyses are reported in the lower right corner of the graph. N) Correlation analysis between ETV7 and TNFRSF1A mRNA level in the whole cohort of BC patients from University Medical Center Hamburg-Eppendorf.

Whole panel: * p≤0.05; ** p≤0.01; *** p≤0.001.

**Supplementary Figure 2.** A) A schematic structure of the reporter plasmid was used to study the NF-κB-dependent transcriptional activity. B-E) RT-qPCR analysis of the known NF-κB target genes A20 (D), IL-6 (E), IL-8 (C), TNF-α (B) in T47D ETV7 or Empty cells treated with TNF-α (15 ng/ml) for 4 hours. Bars represent the averages and standard deviations of at least three biological replicates. F-H) RT-qPCR analysis of the normalized expression of NF-κB target genes in T47D, MDA-MB-231, and SK-BR-3 parental cells transfected with ETV7 targeting siRNA #1 and siRNA #2 or the scrambled control. Bars represent the averages and standard deviations of at least three biological replicates. I) On the left, western blot analysis of phosphorylated IκBα in T47D Empty and ETV7 cells in response to 15 ng/ml TNF-α treatment for 1 hour. The approximate observed molecular weight is indicated on the right side of each blot . HSP70 was used as a loading control. On the right, the quantification of Western blot analysis of phosphorylated IκBα in T47D Empty and ETV7 cells in response to 15 ng/ml TNF-α treatment for 1 hour. Bars represent the averages and standard deviations of at least three biological replicates. Data is normalized to the signal of HSP70, which was used as a loading control. J) Immunofluorescence analysis for p65 (green signal) nuclear translocation in T47D Empty and T47D ETV7 cells. Nuclei are stained in blue. T47D cells were left untreated or stimulated with 15 ng/ml TNF-α for 60 min. Shown are representative data for at least three biological replicates. Images were acquired with a 20X magnification. The arrows indicate the nuclear localization of p65. K) Quantification of nuclear:cytoplasmic ratios of p65 fluorescence intensity in T47D Empty and T47D ETV7 cells. Bars represent mean ± standard deviation from analysis of 10 (per each biological replicate; n=3) separated field images.

Whole panel: * p≤0.05; ** p≤0.01; *** p≤0.001.

**Supplementary Figure 3.** A) A flowchart of the design of the rescue experiment. B) Western blot analysis of the lysates obtained after performing the Luciferase assay (Fig. 4E) to control the over-expression of TNFR1 in MCF7 Empty and ETV7 cells. On the right of each blot is indicated the observed molecular weight. α-actinin was used as a loading control. C-D) RT-qPCR analysis of the expression of IL-6 (D) and IL-8 (C) in the MCF7 Empty and MCF7 ETV7 cells over-expressed with pcDNA3.1-TNFR1 or pcDNA3.1-Empty plasmids and untreated or stimulated with TNF-α (10 ng/ml) for 4 hours. Bars represent the averages and standard deviations of two biological replicates. E) The secretion of TNF-α in the supernatant of MCF-7 and T47D Empty or ETV7 cells at a basal level, measured by ELISA. Bars represent the mean and the standard deviation of three biological replicates. F) The secretion of IL-8 and IL-6 in the supernatant of T47D Empty and T47D ETV7 cells without or with stimulation with TNF-α (15 ng/ml), measured by ELISA. Bars represent the mean and the standard deviation of three biological replicates. G) Dot plots demonstrating the differential expression analysis for IL-6, IL-8, TNFAIP3 (A20), and CCL2 genes in a BRCA-matched patients’ dataset from TCGA (The Cancer Genome Atlas) database. Tumor (light red), Normal (light blue). G) Kaplan–Meier curves for TCGA breast cancer patients stratified according to the average expression of INFLAMMATORY_RESPONSE gene signature. Curves represent the probability of disease-specific survival (DSS). p-values are calculated with the log-rank test. H) INFLAMMATORY_RESPONSE gene set expression in TCGA BRCA samples classified by PAM50 molecular subtypes. p-values are shown when significant.

Whole panel: * p≤0.05; ** p≤0.01; *** p≤0.001.

**Supplementary Figure 4.** A) Gene Set Enrichment Analysis of T47D cells over-expressing ETV7 or its Empty counterpart: Shown is the Enrichment plot for IL6_JAK_STAT3 signaling in T47D cells gene sets of the Hallmark Collection. The Normalized Enrichment Score (NES) shows the degree of the enrichment of the gene set; the negative sign indicates that the gene set is down-regulated in cells over-expressing ETV7. FDR = False Discovery Rate. B) The quantification of Western blot analysis of pSTAT3 in MCF7 Empty and ETV7 cells (Fig. 5C). Bars represent the averages and standard deviations of at least three biological replicates. Data is normalized to the signal of histone H3, which was used as a loading control. C) RT-qPCR analysis for the expression of TNFRSF1A after the stimulation with IL-6 (20 ng/ml) for 4 hours in MCF7 cells over-expressing ETV7 or its empty counterpart. D) Western blot analysis of subcellular fractionation from T47D Empty and T47D ETV7 cells untreated or treated with IL-6 (20 ng/ml) for 4 hours. On the right of each blot is indicated the approximate observed molecular weight. Cyt – cytoplasmic protein fraction, Chr – chromatin-enriched protein fraction. GAPDH was used as a loading control for the cytoplasmic fraction. Histone H3 was used as a loading control for chromatin-enriched protein fraction. E) Western blot analysis of the lysates from ChIP assay to control the phosphorylation of STAT3. F) RT-qPCR analysis of STAT3 expression in MCF7 or T47D cells over-expressing ETV7 or its Empty counterpart. G) ChIP-qPCR of TNSFRSF1A Intron 1 Binding site #3 in MCF7 cells over-expressing ETV7 untreated or treated with IL-6 (20 ng/ml) for 4 hours. The percentage of the enrichment of pSTAT3 or control (normal rabbit IgG) bound to TNFRSF1A Intron 1 with respect to Input DNA is shown. NSB=non-specific binding, a region within the *ACTB* promoter. BS = binding site. Bars represent the averages and standard deviations of at least three biological replicates. H) ChIP-qPCR analysis of the regulatory region of MED16 in MCF7 cells over-expressing ETV7 or its empty counterparts. The percentage of the enrichment of pSTAT3 or control (normal rabbit IgG) bound to MED16 regulatory region with respect to Input DNA is shown. Bars represent the averages and standard deviations of at least three biological replicates. I-J) ChIP-qPCR analysis of the Intron I of TNFRSF1A binding site #1 and binding site #2 in MCF7 (I) and T47D (J) parental cells untreated or treated with 1.5 µM Doxorubicin (24 h), 20 ng/ml IL-6 (4 h), or the combination (IL-6+Doxo). The percentage of the enrichment of pSTAT3 or control (normal rabbit IgG) bound to TNFRSF1A Intron 1 with respect to Input DNA is shown. BS = binding site. Bars represent the averages and standard deviations of at least three biological replicates. K) Co-immunoprecipitation analysis of protein-protein interaction between ETV7 and pSTAT3 in MCF7 and T47D cells over-expressing ETV7. Normal IgG and INPUT were used as controls. Whole panel: n.s. – not significant; * p≤0.05; ** p≤0.01; *** p≤0.001.
