# Supplementary material for: ETV7 reduces inflammatory responses in breast cancer cells by repressing the TNFR1/NF-κB axis
Source: Cell Death Dis. 2023 Apr 12;14(4):263. doi: 10.1038/s41419-023-05718-y (PMC10089821; doi:10.1038/s41419-023-05718-y)
Supplement: Supplementary file 7 — Supplementary Table 1 [file 41419_2023_5718_MOESM7_ESM.docx]

**Supplementary Table 1. The sequence of the primers used in the study.**

| **RT-qPCR Primer** | **Sequence 5’→3’** |
| --- | --- |
| ETV7-Fw | CAAGATCTTCCGAGTTGTGGA |
| ETV7-Rv | GTTCACCCGGTTCTTGTGAT |
| IL1R1-Fw | CTGAGAAGCTGGACCCCTTG |
| IL1R1-Rv | GCATTTATCAGCCTCCAGAGA |
| IL10RB-Fw | GTGAGCCTGTCTGTGAGCAA |
| IL10RB-Rv | TGAGGATGGCCCAAAAACTCTT |
| ACTB-Fw | AAGAATTCATACCGCCGAGACCGCGTCCGC |
| ACTB-Rv | AACATATGGGTGAGCTGGCGGCGGGTGT' |
| YWHAZ-Fw | CAACACATCCTATCAGACTGGG |
| YWHAZ-Rv | AATGTATCAAGTTCAGCAATGGC |
| IL6-Fw | GAAAGCAGCAAAGAGGCACT |
| IL6-Rv | TTTCACCAGGCAAGTCTCCT |
| IL8-Fw | CTCTCTTGGCAGCCTTCCT |
| IL8-Rv | GGGTGGAAAGGTTTGGAGTA |
| A20 (TNFAIP3)-Fw | AAAGCCCTCATCGACAGAAA |
| A20 (TNFAIP3)-Rv | AGAAGTGGCATGCATGAGG |
| TNF-α Fw | GGGACCTCTCTCTAATCAGC |
| TNF-α Rv | TCAGCTTGAGGGTTTGCTAC |
| TLR2-Fw | GCCTCTCCAAGGAAGAATCC |
| TLR2-Rv | TCCTGTTGTTGGACAGGTCA |
| STAT3-Fw | GAAACAGTTGGGACCCCTGA |
| STAT3-Rv | AGGTACCGTGTGTCAAGCTG |
| TNFRSF1A-Fw | ATTGGACTGGTCCCTCACCT |
| TNFRSF1A-Rv | AGTAGGTTCCTTTGTGGCACTT |
| MED16-Fw | GAAAGTGCTCGTTGTTCTACC |
| MED16-Rv | TTGCATACGACCATTTCCAG |
| **ChIP primer** | **Sequence 5’→3’** |
| ACTB (NSB)-Fw | TCTCCCTCCTCCCTTCTTCAAT |
| ACTB (NSB)-Rv | TCGCGCCGCTGGGTTTTATA |
| TNFRSF1A-BS#1-Fw | CACAGACCCTTGTCCCACTT |
| TNFRSF1A-BS#1-Rv | GGGAGACTTCCCTTCGGGA |
| TNFRSF1A-BS#2-Fw | GCCACGTGTTCCCTTCTCTT |
| TNFRSF1A-BS#2-Rv | CTAGTTCCCTCTCCCCTCCC |
| TNFRSF1A-BS#3-Fw | GGGGAAAGGAACCACACTTT |
| TNFRSF1A-BS#3-Rv | TCTGCTGAGAACAGGACTGG |
