# Supplementary material for: ETV7 reduces inflammatory responses in breast cancer cells by repressing the TNFR1/NF-κB axis
Source: Cell Death Dis. 2023 Apr 12;14(4):263. doi: 10.1038/s41419-023-05718-y (PMC10089821; doi:10.1038/s41419-023-05718-y)
Supplement: Supplementary file 8 — Supplementary Table 2 [file 41419_2023_5718_MOESM8_ESM.docx]

| **Supplementary Table 2: Hamburg Cohort Patient characteristics (n=197)** | |
| --- | --- |
|  | **n (%)** |
| ***Age (years)*** |  |
| Median | 56.6 |
| Range | 29-94 |
| ***Histological Type*** |  |
| Ductal | 140 (71) |
| Lobular | 31 (16) |
| Others | 22 (11) |
| Unknown | 4 (2) |
| ***Tumor Size (stage)*** |  |
| < 2cm (pT1) | 51 (26) |
| 2-5cm (pT2) | 121 (61) |
| > 5cm (pT3-4) | 20 (10) |
| Unknown | 5 (3) |
| ***Grade*** |  |
| I | 20 (10) |
| II | 80 (41) |
| III-Undifferentiated | 92 (47) |
| Unknown | 5 (3) |
| ***Lymph Nodes*** |  |
| Positive nodes | 136 (69) |
| Negative nodes | 60 (31) |
| Unknown | 1 (0.5) |
| ***ER Status*** |  |
| Positive | 148 (75) |
| Negative | 41 (21) |
| Unknown | 8 (4) |
| ***PR Status*** | |
| Positive | 124 (63) |
| Negative | 65 (33) |
| Unknown | 8 (4) |
|  |  |
| **Molecular Subtype** |  |
| Luminal | 123 (63) |
| HER2 + | 29 (15) |
| TNBC | 37 (19) |
| Unknown | 8 (4) |
|  |  |
| ***Follow-up*** |  |
| Recurrence | 72 (37) |
| No recurrence | 105 (53) |
| Recurence unknown | 20 (10) |
| Died of disease | 56 (28) |
| Died of other course | 8 (4) |
| Alive | 126 (64) |
| Unknown | 7 (4) |
| Median follow-up period (months) | 133 |
