# Supplementary material for: ETV7 reduces inflammatory responses in breast cancer cells by repressing the TNFR1/NF-κB axis
Source: Cell Death Dis. 2023 Apr 12;14(4):263. doi: 10.1038/s41419-023-05718-y (PMC10089821; doi:10.1038/s41419-023-05718-y)

Fig. 1C

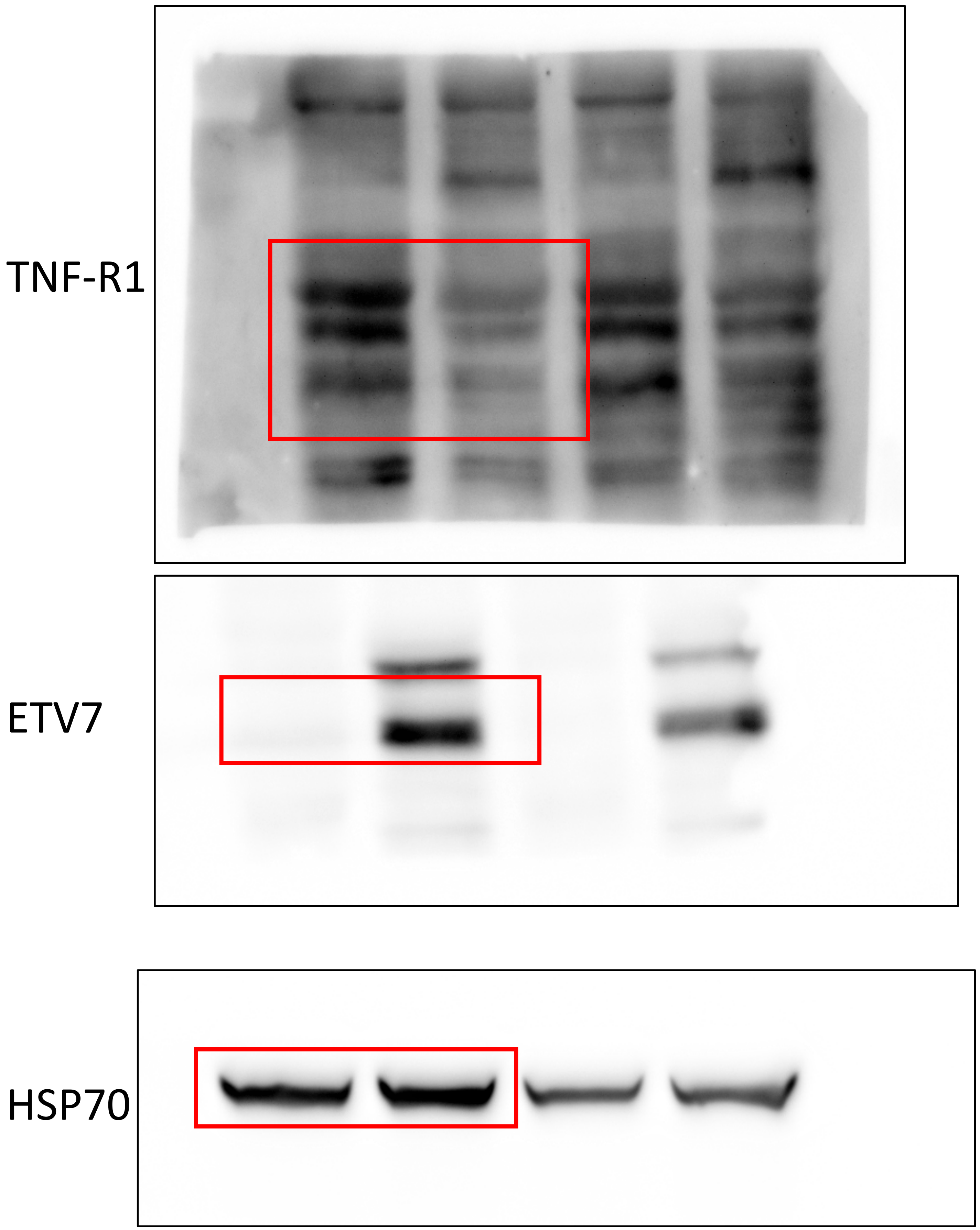

Fig. 1C

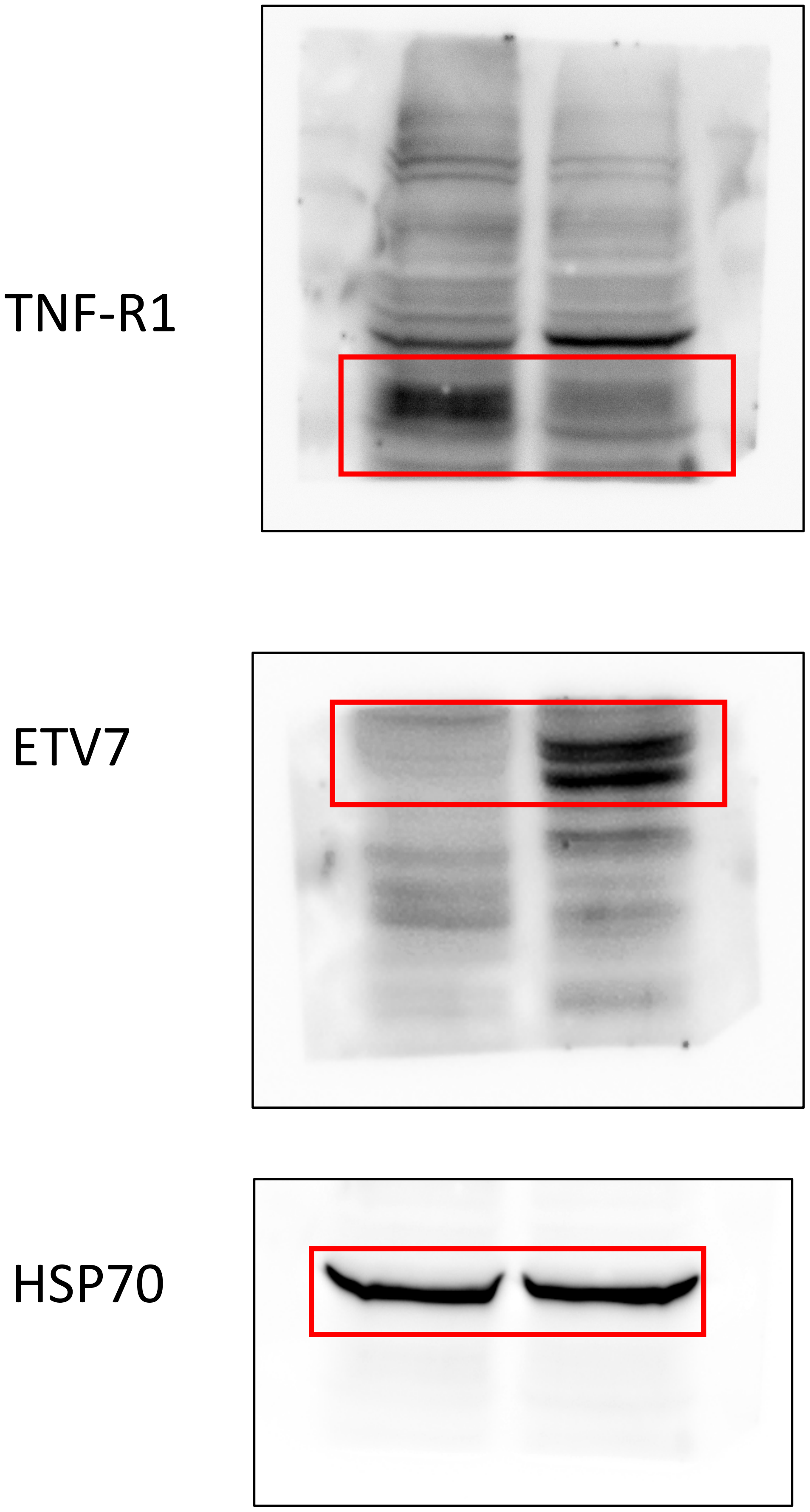

Suppl. Fig. 1B

TNFR1

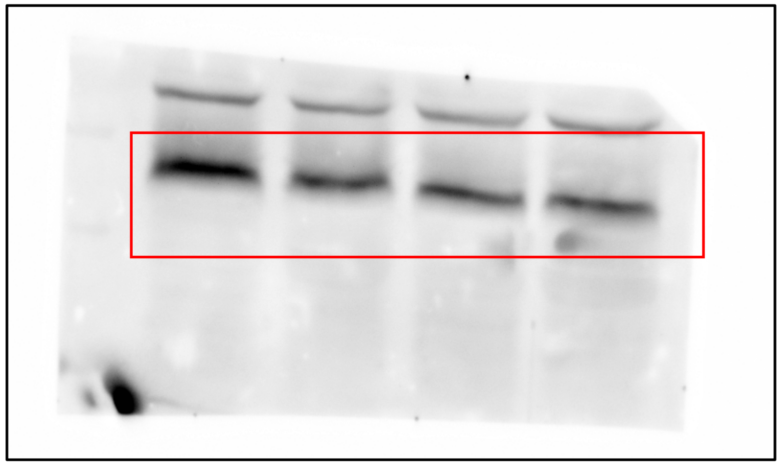

HSP70

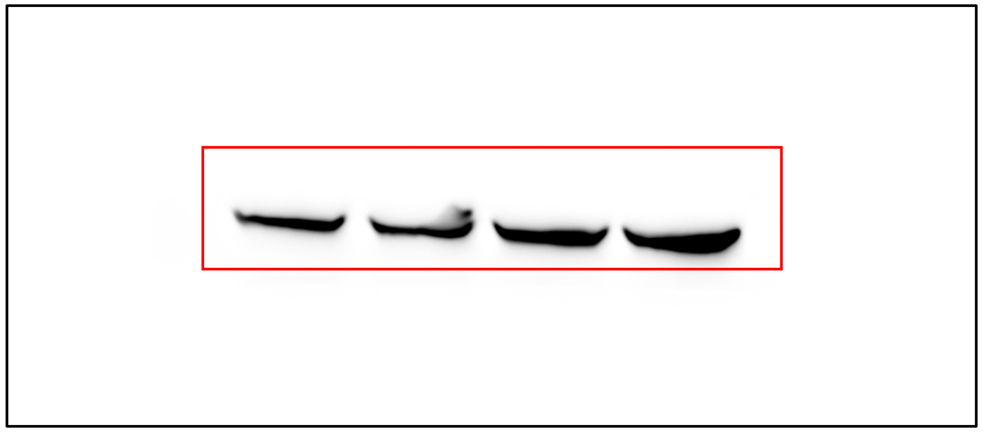

ETV7

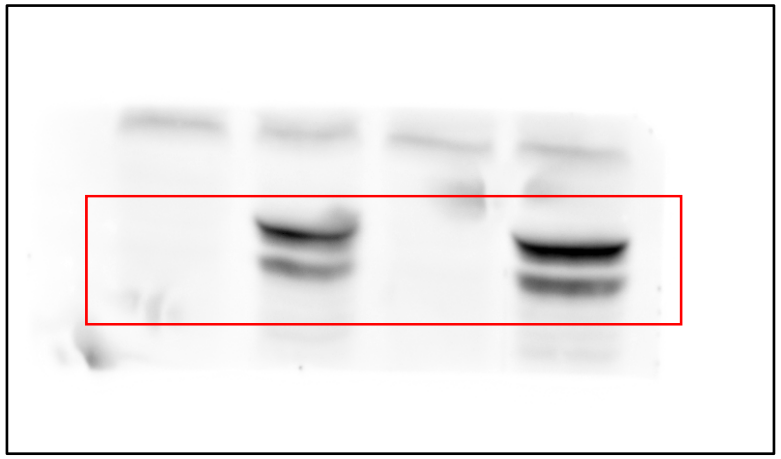

Supplementary fig. 1G

ETV7

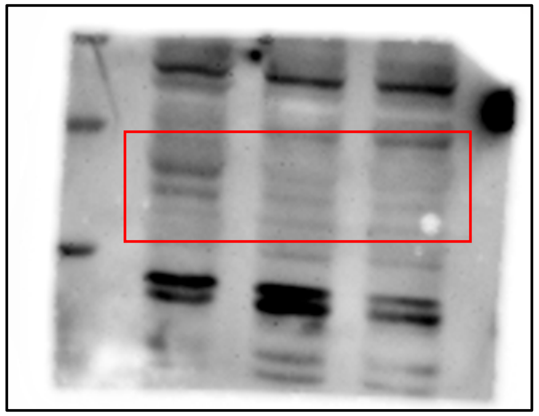

HSP70

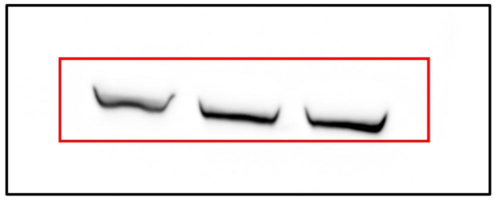

Supplementary fig. 1H

ETV7

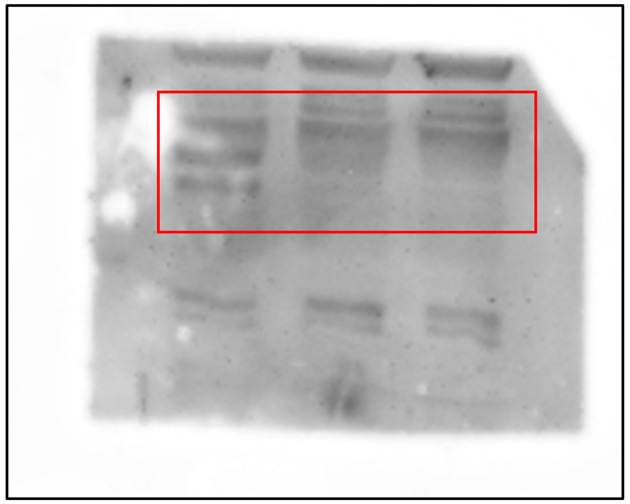

HSP70

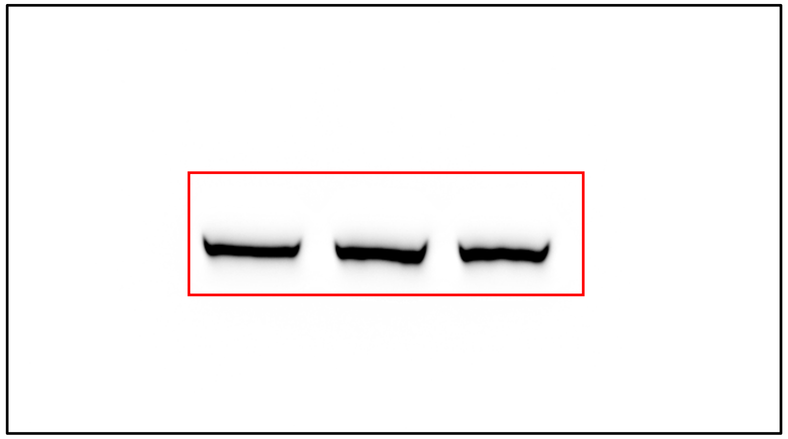

Supplementary fig. 1I

ETV7

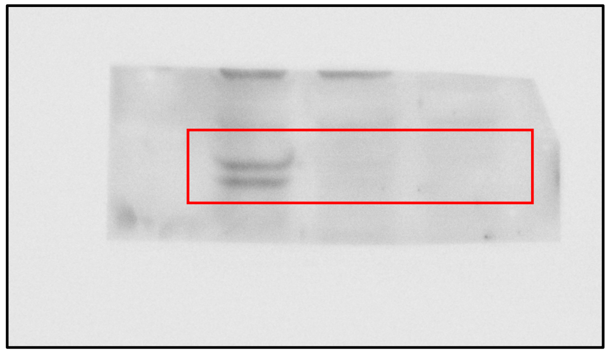

HSP70

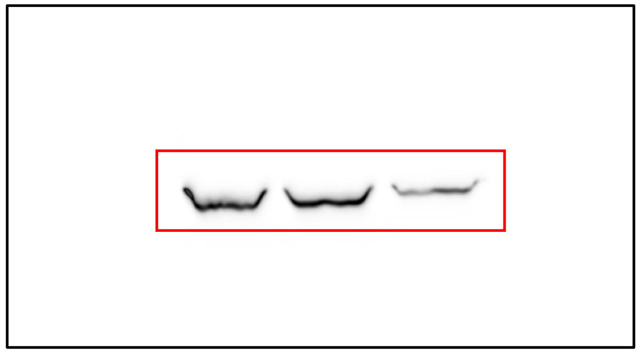

Supplementary fig. 1J

ETV7

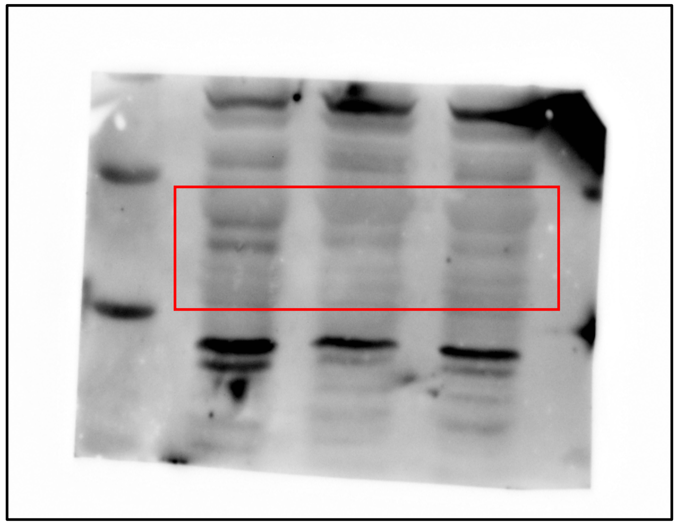

HSP70

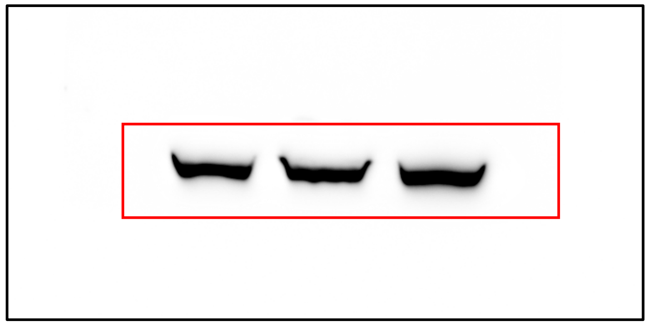

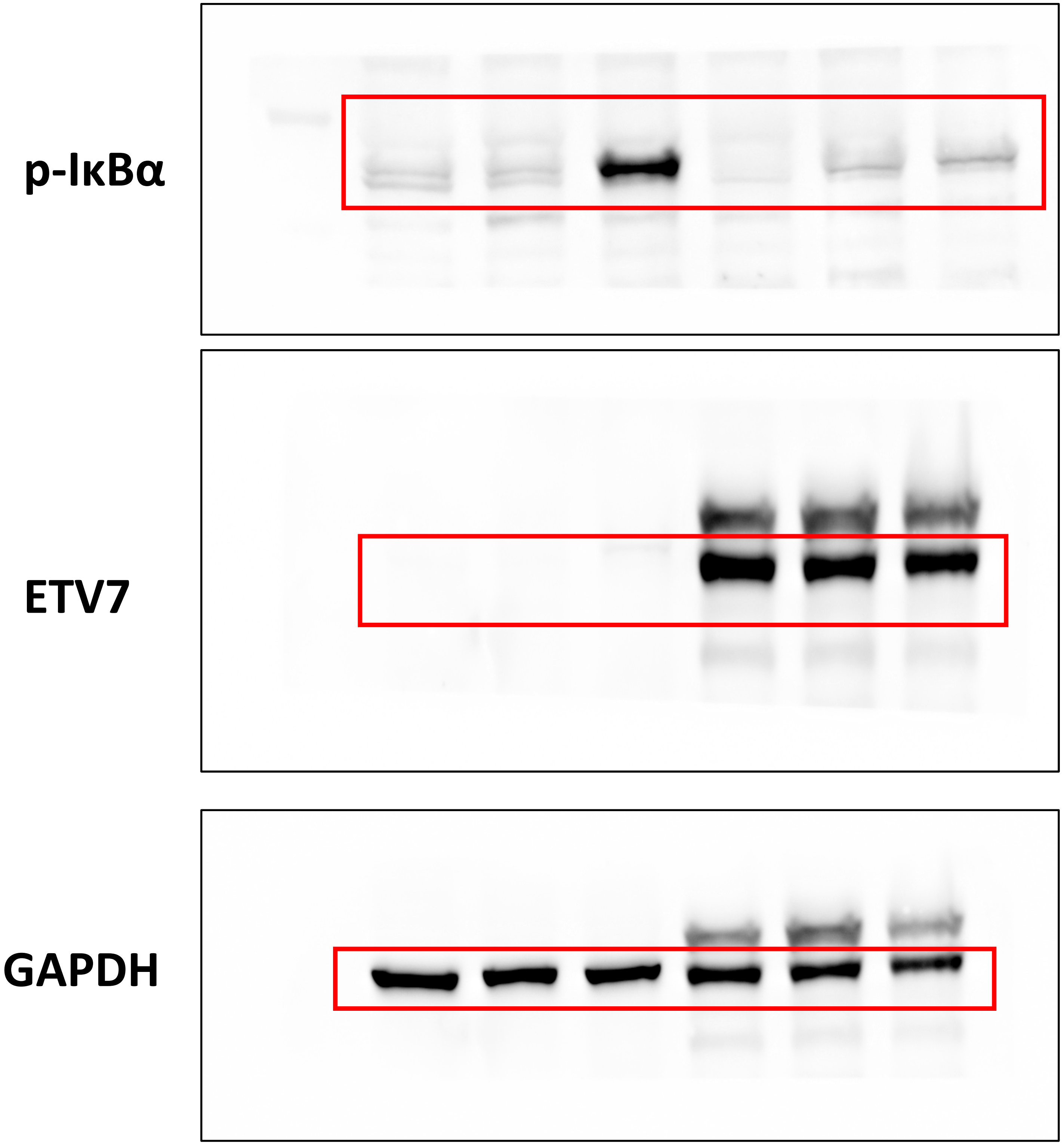
p-IκBα
ETV7
GAPDH

Suppl. Fig. 2I

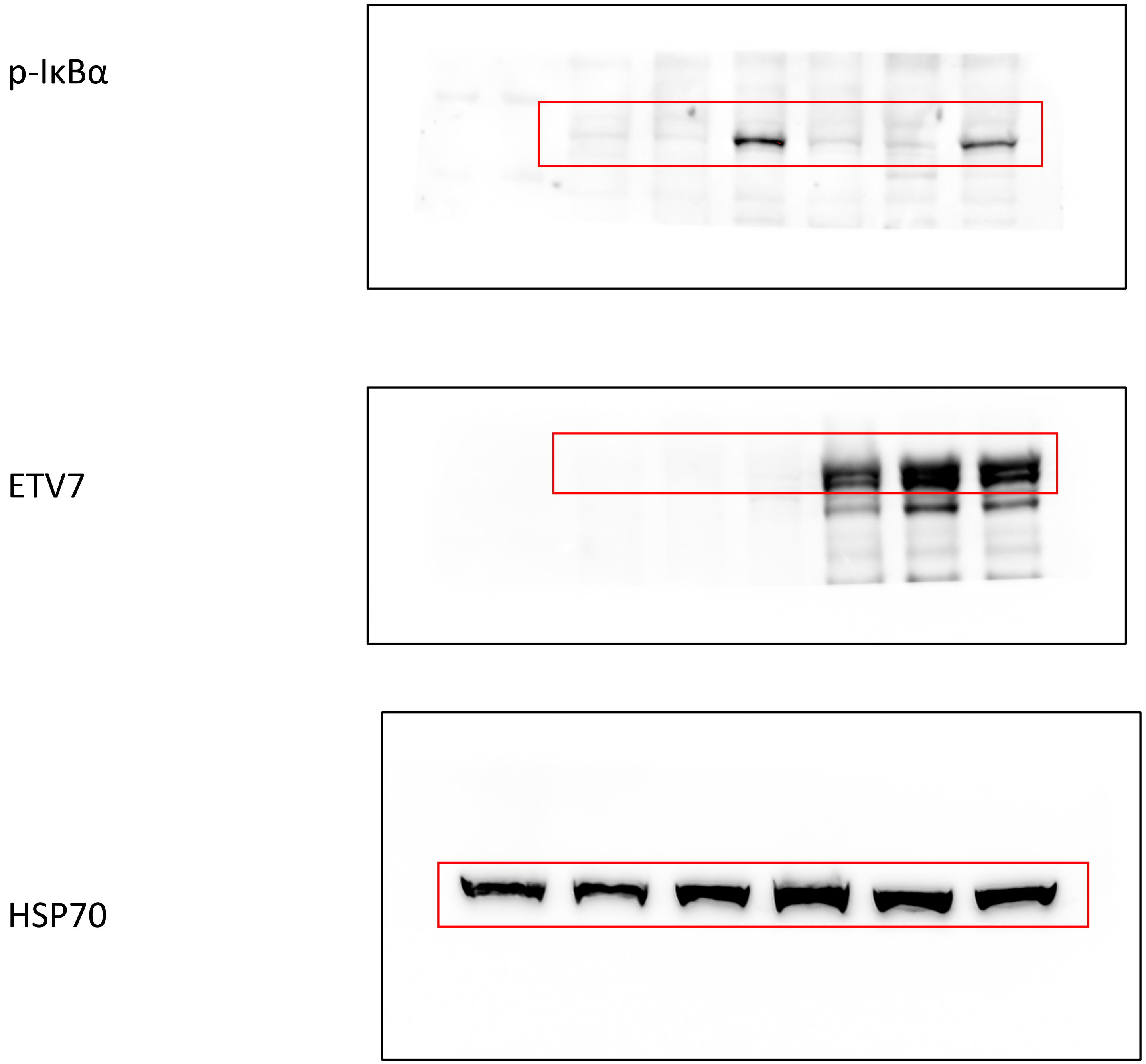

Fig. 5C

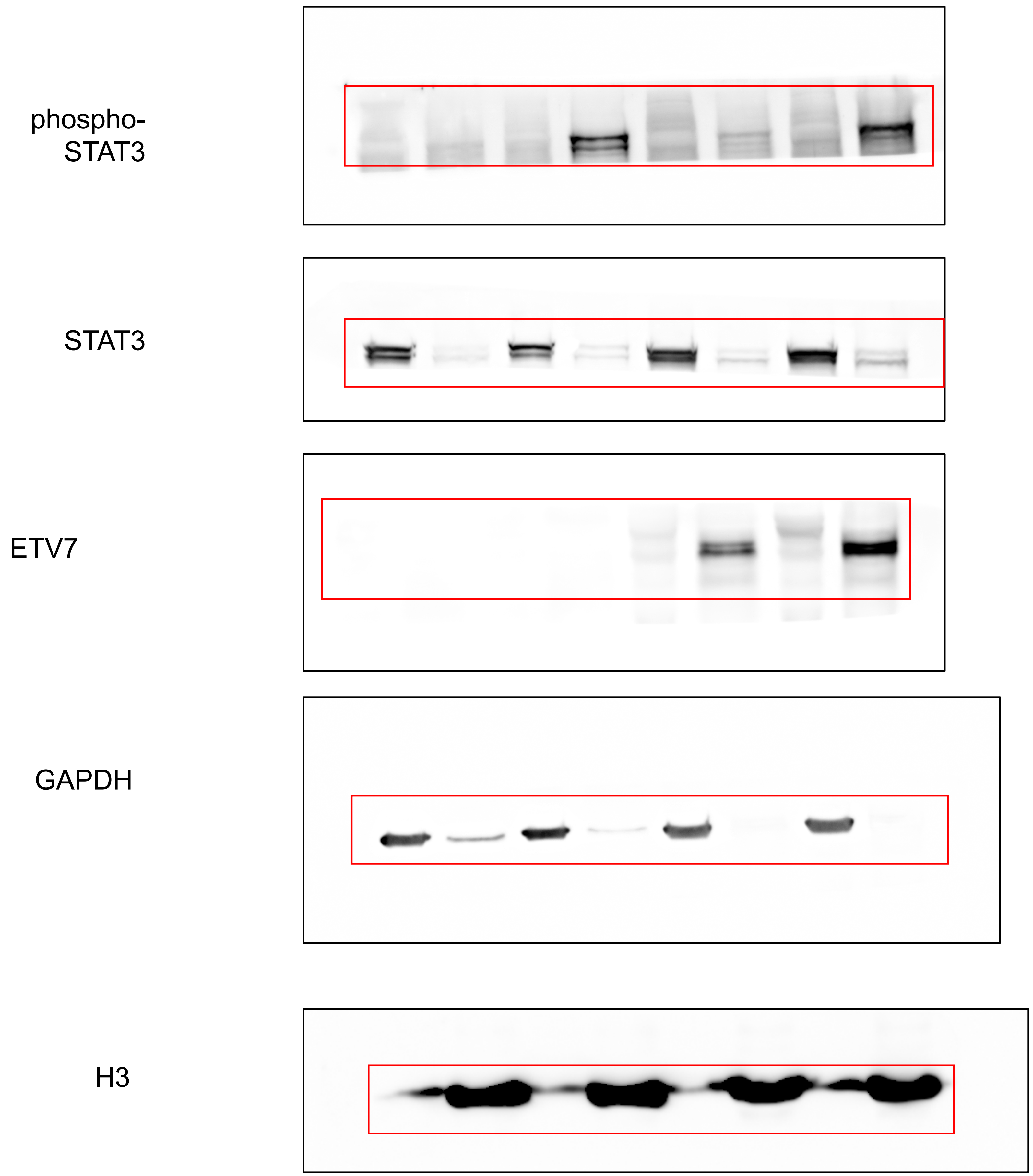

Suppl. Fig. 4D

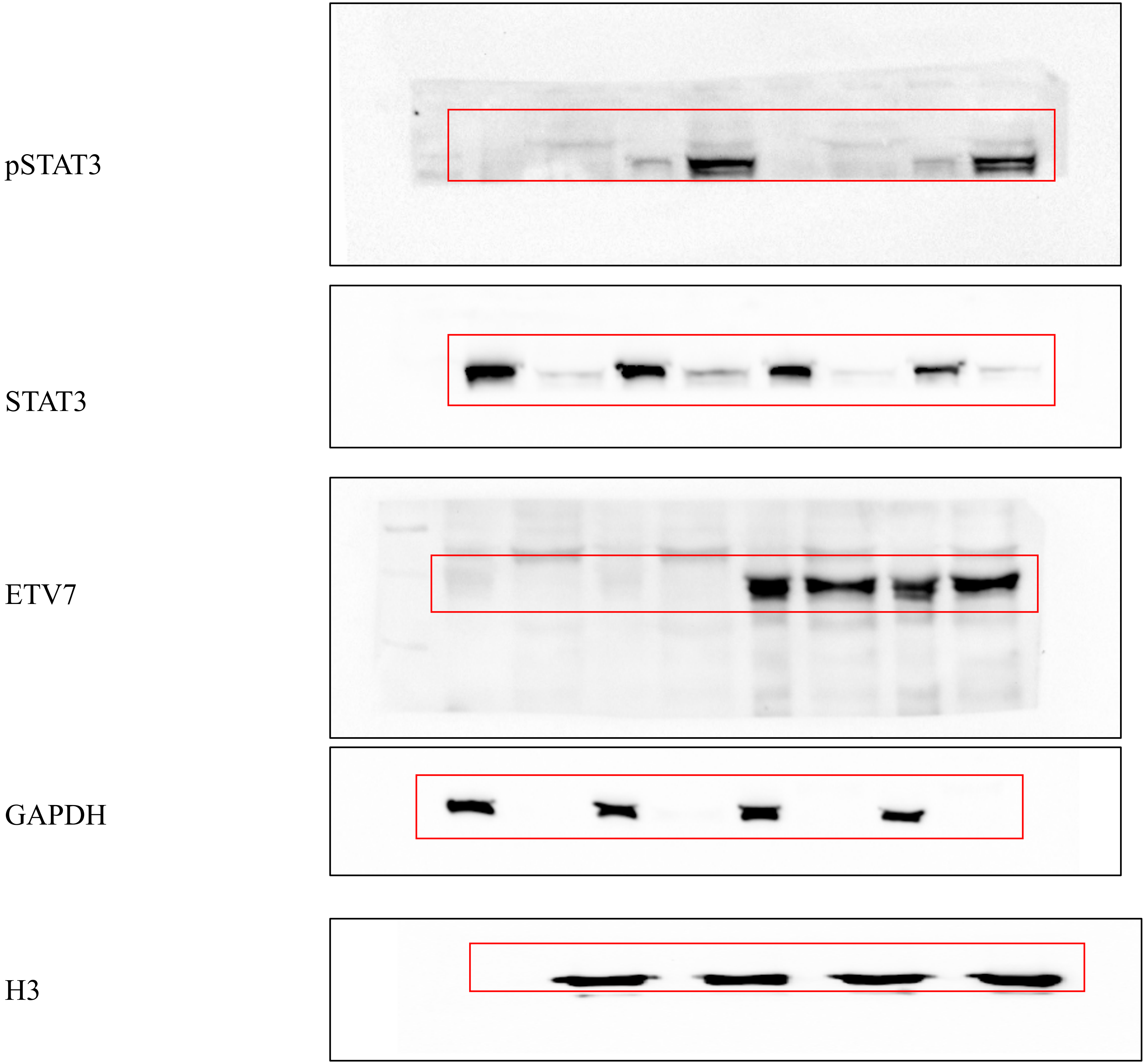

Suppl. Fig. 4E

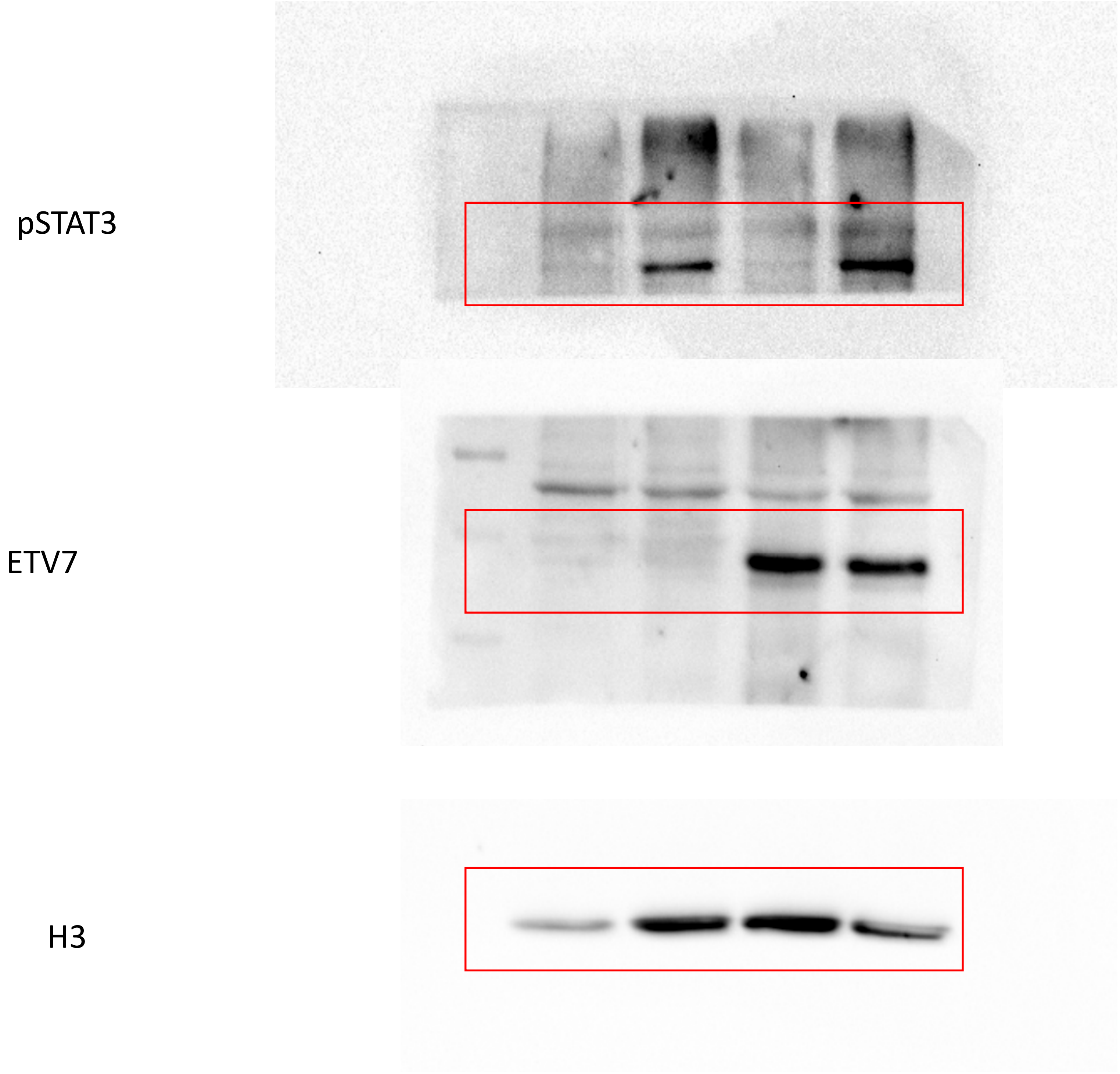

Suppl. Fig. 3B

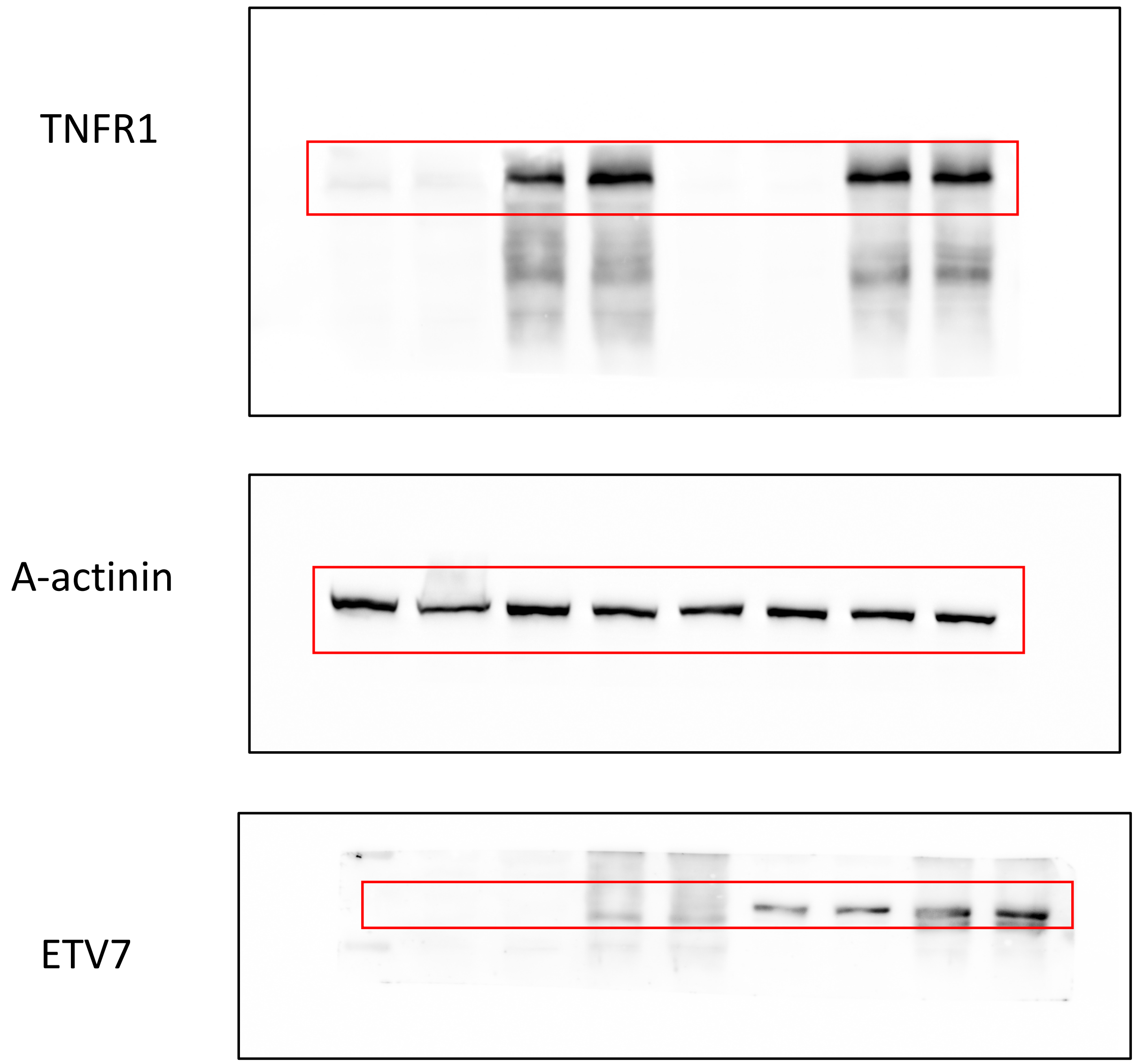

Suppl. Fig. 4 J

pSTAT3

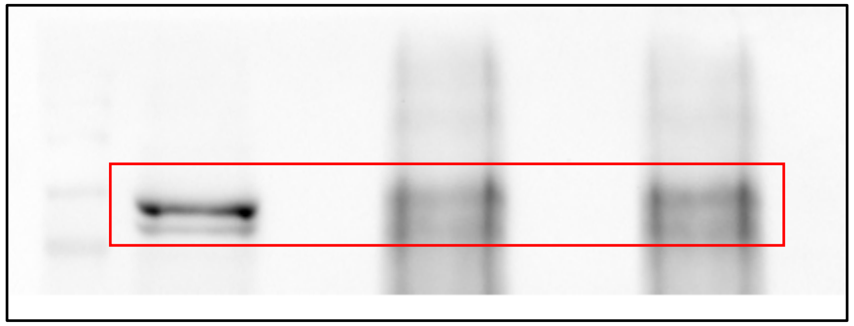

ETV7

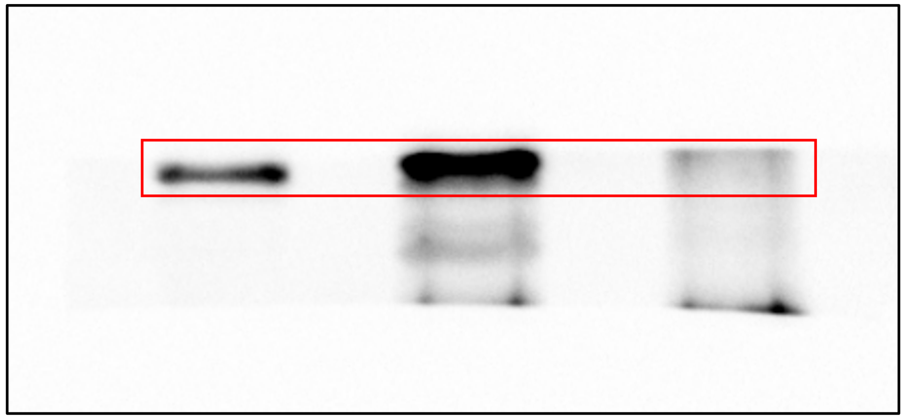

Suppl. Fig. 4 J

pSTAT3

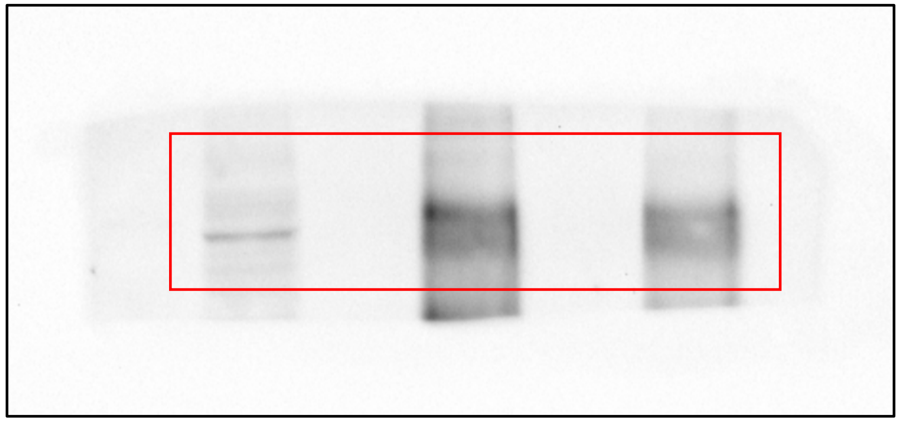

ETV7

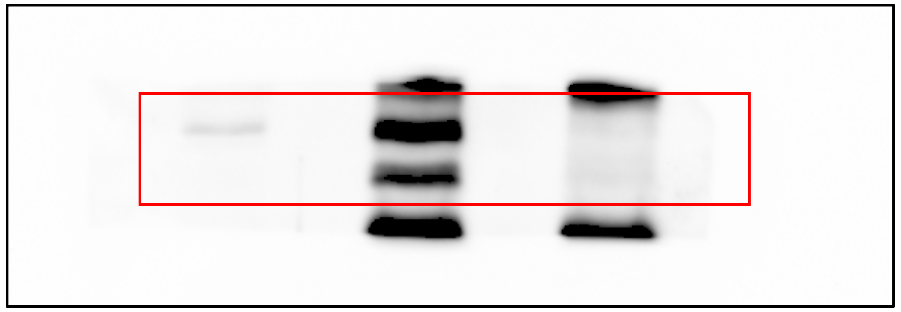

Supplement: Supplementary file 9 — Original WB [file 41419_2023_5718_MOESM9_ESM.pdf]
